# iMAP: an integrated bioinformatics and visualization pipeline for microbiome data analysis

**DOI:** 10.1186/s12859-019-2965-4

**Published:** 2019-07-03

**Authors:** Teresia M. Buza, Triza Tonui, Francesca Stomeo, Christian Tiambo, Robab Katani, Megan Schilling, Beatus Lyimo, Paul Gwakisa, Isabella M. Cattadori, Joram Buza, Vivek Kapur

**Affiliations:** 1 The Huck Institutes of the Life Sciences, Pennsylvania State University, University Park, State College, PA USA; 2 Department of Biochemistry and Molecular Biology, Pennsylvania State University, University Park, State College, PA USA; 3 Biosciences eastern and central Africa-International Livestock Research Institute (BecA-ILRI) Hub, Nairobi, Kenya; 4 Applied Biological and Biosecurity Research Laboratory, Pennsylvania State University, University Park, State College, PA USA; 5 Department of Animal Science, Pennsylvania State University, University Park, State College, PA USA; 6 Nelson Mandela African Institute of Science and Technology, Arusha, Tanzania; 7 Sokoine University of Agriculture, Morogoro, Tanzania; 8 Department of Biology, Pennsylvania State University, University Park, State College, PA USA; 9 Present Address: The European Molecular Biology Laboratory (EMBL), Heidelberg, Germany

**Keywords:** Microbiome bioinformatics, Microbiome data analysis, Microbiome data visualization, Microbial community, Bioinformatics pipeline, 16S rRNA gene, Phylogenetic analysis, Phylogenetic annotation

## Abstract

**Background:**

One of the major challenges facing investigators in the microbiome field is turning large numbers of reads generated by next-generation sequencing (NGS) platforms into biological knowledge. Effective analytical workflows that guarantee reproducibility, repeatability, and result provenance are essential requirements of modern microbiome research. For nearly a decade, several state-of-the-art bioinformatics tools have been developed for understanding microbial communities living in a given sample. However, most of these tools are built with many functions that require an in-depth understanding of their implementation and the choice of additional tools for visualizing the final output. Furthermore, microbiome analysis can be time-consuming and may even require more advanced programming skills which some investigators may be lacking.

**Results:**

We have developed a wrapper named iMAP (Integrated Microbiome Analysis Pipeline) to provide the microbiome research community with a user-friendly and portable tool that integrates bioinformatics analysis and data visualization. The iMAP tool wraps functionalities for metadata profiling, quality control of reads, sequence processing and classification, and diversity analysis of operational taxonomic units. This pipeline is also capable of generating web-based progress reports for enhancing an approach referred to as review-as-you-go (RAYG). For the most part, the profiling of microbial community is done using functionalities implemented in Mothur or QIIME2 platform. Also, it uses different R packages for graphics and R-markdown for generating progress reports. We have used a case study to demonstrate the application of the iMAP pipeline.

**Conclusions:**

The iMAP pipeline integrates several functionalities for better identification of microbial communities present in a given sample. The pipeline performs in-depth quality control that guarantees high-quality results and accurate conclusions. The vibrant visuals produced by the pipeline facilitate a better understanding of the complex and multidimensional microbiome data. The integrated RAYG approach enables the generation of web-based reports, which provides the investigators with the intermediate output that can be reviewed progressively. The intensively analyzed case study set a model for microbiome data analysis.

**Electronic supplementary material:**

The online version of this article (10.1186/s12859-019-2965-4) contains supplementary material, which is available to authorized users.

## Background

Understanding the diversity of microbes living in a given sample is a crucial step that could lead to novel discoveries. The choice of bioinformatics methodology used for analyzing any microbiome dataset from pre-processing of the reads through the final step of the analysis is a key factor for gaining high-quality biological knowledge. Most of the available bioinformatics tools contain multiple functions and may require an in-depth knowledge of their implementation. In most cases, several tools are used independently to analyze a single microbiome dataset and to find the right combination of tools is even more challenging. Obviously, finding suitable tools that complete the analysis of microbiome data can be time-consuming and may even require more high-level programming experiences which some users may be lacking.

The core step in microbiome analysis is the taxonomic classification of the representative sequences and clustering of OTUs (Operational Taxonomic Units). OTUs are pragmatic proxies for potential microbial species represented in a sample. Performing quality control of the sequences prior to taxonomic classification is paramount for the identification of poor-quality reads and residual contamination in the dataset. There are several public tools available for inspecting read quality and filtering the poor-quality reads as well as removing any residue contamination. For example, pre-processing tools such as Seqkit [1], FastQC [2] and BBduk.sh command available in the BBMap package [3] are designed to help investigators review the properties and quality of reads before further downstream analyses. High quality reads coupled with stringent screening and filtering can significantly reduce the number of spurious OTUs.

The most famous microbiome analysis tools integrate different quality control approaches in their pipelines. Mothur [4] for example is well known for its intensive quality filtering of poor sequences before OTU clustering and taxonomy assignment. Quantitative Insight Into Microbial Ecology (QIIME-2), a successor of QIIME-1 [5] (see http://qiime.org/) uses DADA2 [6] to obtain high-quality representative sequences before aligning them using MAFFT [7] software. Nevertheless, the most common sequencing error is the formation of chimeric fragments during PCR amplification process [8, 9]. Briefly, chimeras are false recombinants formed when prematurely terminated fragments during PCR process reanneal to another template DNA, thus eliminating the assumption that an amplified sequence may have originated from a single microbial organism. Detecting and removing chimeric sequences is crucial for obtaining quality sequence classification results. Both Mothur and QIIME-2 integrate special tools for chimera removal, specifically UCHIME [10] and VSEARCH [11].

The sequences that pass the filtering process are typically searched against a known reference taxonomy classifier at a pre-determined threshold. Most classifiers are publicly available including the Ribosomal Database Project (RDP) [12], SILVA [13], Greengenes [14], and EzBioCloud [15]. Use of frequently updated databases avoids mapping the sequences to obsolete taxonomy names. In some cases, users may opt to train their custom classifiers using, for example, q2-feature-classifier protocol [14, 15] available in QIIME-2 [16, 17] or use any other suitable method. Over-classification of the representative sequences can result in spurious OTUs, but this can be avoided by applying stringent cut-offs [18].

Frequently, users adopt the default settings of their preferred pipelines. For example, the 97% threshold typically expressed as 0.03 in Mothur and 70% confidence level expressed as 0.7 in QIIME-2 are default settings in OTU clustering. The final output of most microbiome analysis pipelines is the OTU table. Typically, the OTU table is the primary input for most downstream analyses leading to alpha and beta diversity information in both Mothur and QIIME. The OTU table is typically a matrix of counts of sequences, OTUs or taxa on a per-sample basis. The quality of data in the OTU table depends primarily on the previous analyses which provide input to the pipeline’s subsequent steps. Making biological conclusions from the OTU table alone without reviewing the intermediate output is a high risk that could result in inaccurate conclusions.

In the present paper, we developed an improved microbiome analysis pipeline named iMAP (Integrated Microbiome Analysis Pipeline) that integrates exploratory analysis, bioinformatics analysis, intensive visualization of intermediate and final output, and phylogenetic tree annotation. The implementation of iMAP pipeline is demonstrated using a case study where 360 mouse gut samples are intensively analyzed. Throughout the manuscript, “iMAP pipeline” terminology is used instead of “iMAP” for easy readability.

## Methods

### Workflow

**Fig. 1:**
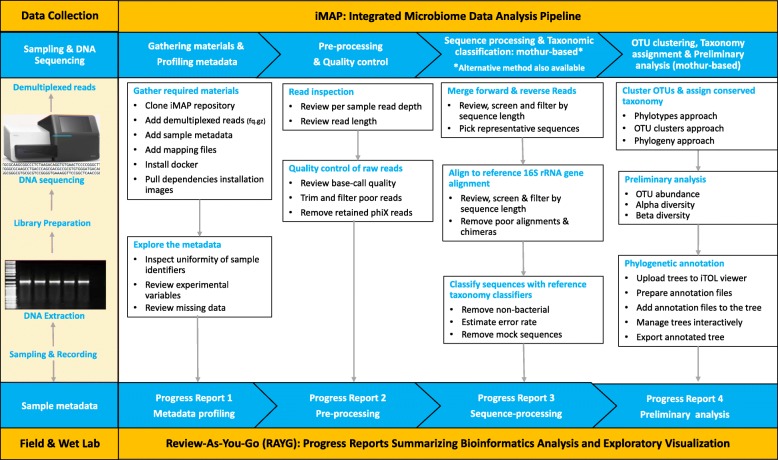
Schematic illustration of the iMAP pipeline. The required materials including data files, software, and reference databases must be in place before executing the iMAP pipeline. The initial step in the analysis is sample metadata profiling followed by pre-processing and quality checking of demultiplexed 16S read pairs which are then merged, aligned to reference alignments, classified then assigned conserved taxonomy names. Output from each major step is transformed, visualized, and summarized into a progress report

Code for implementing iMAP pipeline contains bundles of commands wrapped individually in driver scripts for performing exploratory analysis, preprocessing of the reads, sequence processing and classification, OTU clustering and taxonomy assignment, and preliminary analysis, and visualization of microbiome data (Fig. 1). The pipeline transforms the output obtained from major analysis steps to provide data structure suitable for conducting exploratory visualization and generating progress reports.

### Implementation

A detailed guideline for implementing iMAP pipeline is in the README file included in the iMAP repository. It is mandatory that all user data files are placed in the designated folders and must remain unaltered throughout the entire analysis.

### Robustness, reproducibility, and sustainability

Ability to reproduce microbiome data analysis is crucial. Challenges in robustness and reproducibility are accelerated by lack of proper experimental design, the complexity of experiments, constant updates made to the available pipelines, lack of well-documented workflows, and relying on inaccessible or out-of-date codes. The pre-release version of iMAP described in this manuscript (iMAP v1.0) is at the preliminary phase, and perhaps it lacks significant reproducibility aspects compared to the modern bioinformatics workflow management systems such as Nextflow [19], NextflowWorkbench [20], or Snakemake [21]. In its current state, users will be able to follow the guideline presented in the README file and reuse the associated code interactively, including nested bash and visualization scripts to realize similar results. In an effort to ensuring that the iMAP pipeline is reproducible, portable, and shareable, we created Docker images that wrangle the dependencies including software installation and different versions of R packages. Using Docker images makes it easier for users to deploy the iMAP and run all analyses using containers. Instructions on how to work with Docker are available in the README file. The iMAP pipeline also comes with both mothur and QIIME2 Docker images for the classification of the 16S rRNA gene sequences.

Future sustainability and reproducibility of iMAP depend highly on the use of a well-established workflow management system to provide a fast and comfortable execution environment, which will probably increase the usability as well. A long-term goal is to automate most of the interactive steps and integrate the pipeline with a code that defines rules for deploying across multiple platforms without any modifications.

### Bioinformatics analysis

The iMAP pipeline is intended to be executed interactively from a command line interface (CLI) or from a Docker container CLI to optimize user interaction with the generated output. A detailed guideline is provided in the README file of the iMAP pipeline. Most of the analysis run at default settings unless altered by the user. By default, the iMAP pipeline uses up-to-date SILVA seed classifiers [13] for mothur-based taxonomy assignments or Greengenes classifiers [14] if using QIIME2 pipeline. The SILVA seed and Greengenes databases are relatively small compared to the SILVA NR version which is available for both mothur and QIIME2. Users need to be aware that the larger a dataset is, the more memory (RAM) the system requires. Users may opt to use their preferred classifiers and make a small modification in the sequence classification script. Instructions to do so are available in the README file. We are aware that some microbiome experiments do sequence a mock community to help in measuring the error rate due to biases introduced in PCR amplification and sequencing. The mock community sequences are removed automatically before OTU clustering and taxonomy assignment. However, the group name(s) of the mock samples is required. By default, the iMAP removes two groups named Mock and Mock2. Instruction to replace the mock group names is available in the README.

### Data transformation and preliminary analysis

The final output of most microbiome analysis pipelines is the OTU table, which is typically a matrix of counts of sequences, the observations, i.e. OTUs or taxa on a per-sample basis. The OTU tables are transformed into data structures suitable for further analysis and visualization with R [22]. Most of the analyses and visualization are executed via the RStudio IDE (integrated development environment) [23]. We understand that different investigators prefer different analysis types based on the hypotheses under question. In the following section, we used a case study to demonstrate the application of the iMAP pipeline and the exploratory visualization that provides an insight into the results.

### Phylogenetic annotation with iTOL

We specifically chose a phylogenetic-based annotation with iTOL (integrative Tree Of Life) [24] to be part of the iMAP pipeline as a model for displaying multivariate data in easily interpretable ways. Briefly, phylogenetic annotation of the groups (samples) or taxa requires a pre-built tree such as Newick tree. Fortunately, in both mothur and QIIME2, there are methods for producing Newick trees where samples are clustered using the UPGMA (Unweighted Pair Group Method with Arithmetic Mean). The annotation is done interactively by first uploading the tree into the iTOL tree viewer and then adding prepared plain text annotation files on top of the tree. We advise users to get an overview of different videos, tutorials, and functions available at the iTOL site to understand the details involved in the annotation process.

## Application

### Reproducible case study

Here we use a case study to demonstrate step-by-step how to use iMAP to analyze microbiome data. We use a dataset from a published microbiome study to demonstrate the implementation of the iMAP pipeline. Using published data enables users to see the added value, such as the metadata profiling, preprocessing of reads, extended visualization, and generation of the progress report at every major analysis step. Review of these reports facilitates making an informed decision on whether to proceed or terminate the analysis and make more changes to the experiment.

### Preamble

In 2012 Schloss et al. [25] published a paper in Gut Microbes journal entitled “Stabilization of the murine gut microbiome following weaning”. In this study, 360 fecal samples were collected from 12 mice (6 female and 6 male) at 35 time points throughout the first year. Two mock community samples were added in the analysis for estimating the error rate. The mouse gut dataset was chosen because it has been successfully used in several studies for testing new protocols and workflows related to microbiome data analysis [26, 27].

### Raw data

The demultiplexed paired-end 16S rRNA gene reads generated using Illumina’s MiSeq platform were downloaded from http://www.mothur.org/MiSeqDevelopmentData/StabilityNoMetaG.tar. These reads were the result of amplification of region four (V4) of the 16S rRNA gene. Sample metadata file describing the major features of the experiment and the associated variables was manually prepared. Mapping files, that link paired-end sequences with the samples and design files that linked sample identifiers to individual experimental variables were extracted from the metadata file in a format compatible with Mothur (Additional file 1). Installation of software and download of required reference databases was done automatically. All required materials were placed in the designated folders precisely as described in the guideline and verified using a check file script.

### Metadata profiling

Metadata profiling was done as part of exploratory analysis to specifically explore the experimental variables to help in planning the downstream analysis and find out if there were any issues such as missing data. The sample identifiers were inspected and uniformly coded to facilitate sorting across multiple analytical platforms and for better visualization and uniform labeling of the axes.

### Sequence pre-processing and quality control

Read pre-preprocessing included (i) general inspection using seqkit [1] software to provide basic descriptive information about the reads including data type (DNA or RNA), read depth and read length, (ii) assessing the base call quality using FastQC [2] software, and (iii) trimming and filtering poor reads and removing any retained phiX control reads using BBDuk tool from BBMap [28] package. The quality of altered reads was again verified by re-running the FastQC software. The FastQC output was summarized using MultiQC [29] software.

### Sequence processing and classification

This case study uses mothur-based functions to process and classify the representative sequences. The iMAP code also includes a batch script for analyzing the sequences using QIIME2. Preprocessed paired-end reads were merged into more extended sequences then screened to match the targeted V4 region of the 16S rRNA gene. The pipeline generated the representative sequences and aligned them to the SILVA-seed v132 rRNA reference alignments [30] to find the closest candidates. Post-alignment quality control involved repeating the screening and filtering the output by length and removal of poor alignments and chimeric sequences. All non-chimeric sequences were searched against SILVA-seed classifiers at 80% identity using a k-nearest neighbor consensus and Wang approach precisely as described in the Mothur MiSeq SOP tutorial [24].

Additional quality control was done automatically using ‘remove.lineage’ function run within mothur to remove any non-bacterial or unknown sequences before further analysis. Briefly, by default, the pipeline classified the sequences using SILVA seed taxonomy classifier. If the classifier did not find a match in the database, it grouped the unclassified sequences into ‘unknown’ category. The iMAP code was set to remove all undesirable matches including the unknown and any sequences classified to non-bacterial lineages such as eukaryotes, chloroplast, mitochondria, viruses, viroid and archaea. The sequencing error rate was then estimated using sequences from the mock community. Finally, after error rate estimation all mock sequences were removed from further analysis.

### OTU clustering and conserved taxonomy assignment

We used a combination of phylotype, OTU-based and phylogeny methods to assign conserved taxonomy to OTUs. Briefly, in phylotype method, the sequences were binned into known phylotypes up to genus level. In the OTU-based method, all sequences were binned into clusters of OTUs based on their similarity at ≥97% identity, and precision and FDR were calculated using the opticlust algorithm, a default mothur function for assigning OTUs. The phylogeny method was used to generate a tree that displayed consensus taxonomy for each node. The output from phylotype, OTU-based and phylogeny methods was manually reviewed, de-duplicated and integrated to form a complete OTU taxonomy output.

### Data transformation and preliminary analysis

We prepared data structures for further analysis and visualization with R packages executed via RStudio IDE. In summary, the preliminary analysis included measuring diversity in community membership using Jaccard dissimilarity coefficients based on the observed and estimated richness. The diversity in community structure across groups was determined using Bray-Curtis dissimilarity coefficients. The Bray-Curtis dissimilarity coefficients were further analyzed using ordination methods to get a deeper insight into the sample-species relationships. Included in the ordination-based analysis were: (i) Principal Component Analysis (PCA), (ii) Principal Coordinate Analysis (PCoA or MDS) and (iii) Non-Metric Dimensional Scaling (NMDS). Scree plot was used to find the best number of axes that explained variation seen on PCA plots while PCoA loadings and goodness function in vegan [31] was used to generate values for plotting observations into ordination space. Shepard plot was used to compare observations from original dissimilarities, ordination distances and fitted values in NMDS.

### Phylogenetic annotation

Phylogenetic annotation was done using iTOL tree viewer [24] interactively. To see how the samples clustered together we uploaded mothur-based Newick tree generated from the Bray-Curtis dissimilarity distances into the iTOL tree viewer. We then added on top of the tree three iTOL-compatible annotation files prepared manually to specifically include selected output, including species richness, diversity and relative abundances at phylum-level.

## Results

### Metadata profiling

**Fig. 2:**
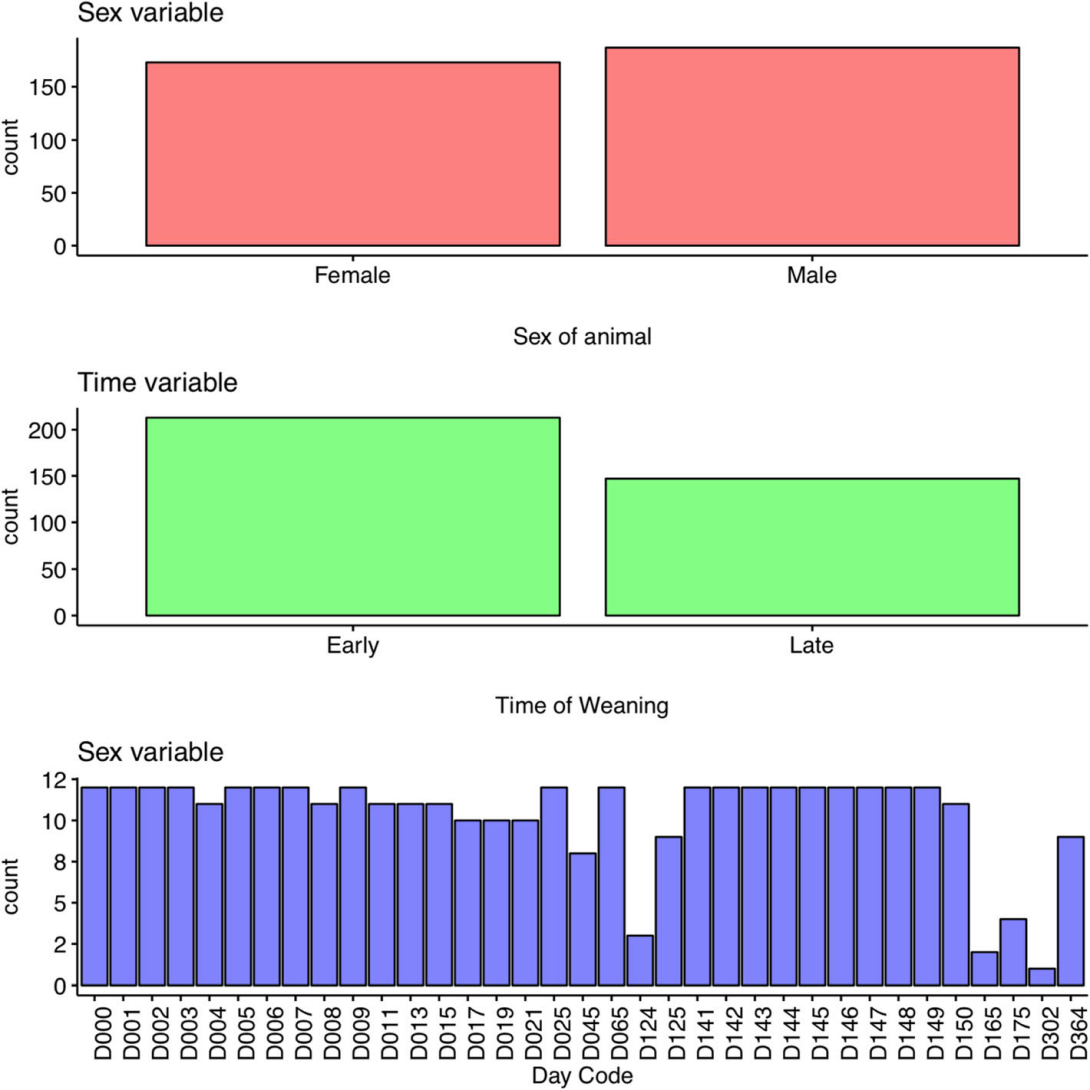
Frequency of categorical variables. The sex and time variables contain two levels each. The days post weaning (DPW) variable contains 35 levels representing data points where D stands for the day the data was collected followed by a numeric value specifying the day number within a year, starting from 0 to 364

Preliminary analysis of the metadata (Additional file 1: Sheet 1) was done to explore the experimental variables and find out any inconsistency or missing values. The results were automatically summarized into a web-based progress report 1 (Additional file 2). The main variables studied were sex (female and male), time range (early and late) grouped based on days-post-weaning (DPW) (Fig. 2). Reviewing the report enabled us to inspect the input data, find the inconsistency in sample coding, and missing data. Before further analysis, the sample identifiers were uniformly re-coded to six figures, e.g., F3D1, F4D11, M4D145 to F3D001, F4D011, M4D145, respectively. In the subsequent analyses, we defined the numeric categoric variables (DPW) as factors and coded it uniformly as shown in DayID column in the metadata file (Table 1).

**Table 1:** Descriptive statistics of the metadata

Variable	q_zeros	p_zeros	q_na	p_na	q_inf	p_inf	type	unique
SampleID	0	0.00	0	0	0	0	character	360
Group	0	0.00	0	0	0	0	character	360
Sex	0	0.00	0	0	0	0	character	2
Time	0	0.00	0	0	0	0	character	2
DPW	12	3.33	0	0	0	0	integer	35
DayID	0	0.00	0	0	0	0	character	35
Description	0	0.00	0	0	0	0	character	1

Key: *q_zeros* quantity of missing data, *p_zeros* percentage of missing data, *q_na* quantity of NA, *p_na* percentage of NA, *q_inf* quantity of infinite values, *p_inf* percentage of infinity values, *type* factor, character, integer or numeric, *unique* frequency of the values

### Read pre-processing and quality control

**Fig. 3:**
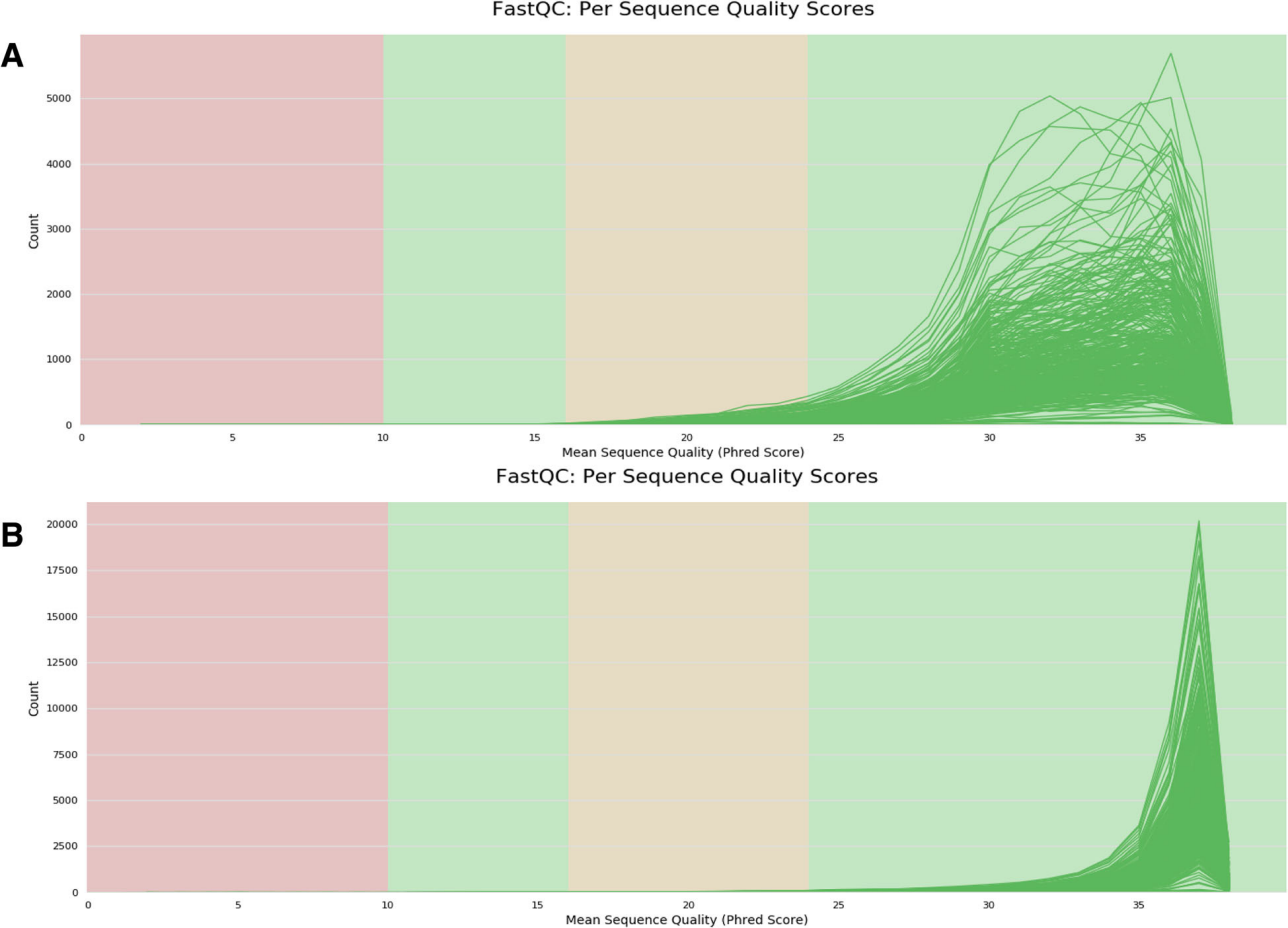
Summary of FastQC quality scores of paired-end reads from 360 samples. The number of reads with average quality scores before (**a**) and after trimming at Q25 and removal of phiX contamination (**b**)

**Fig. 4:**
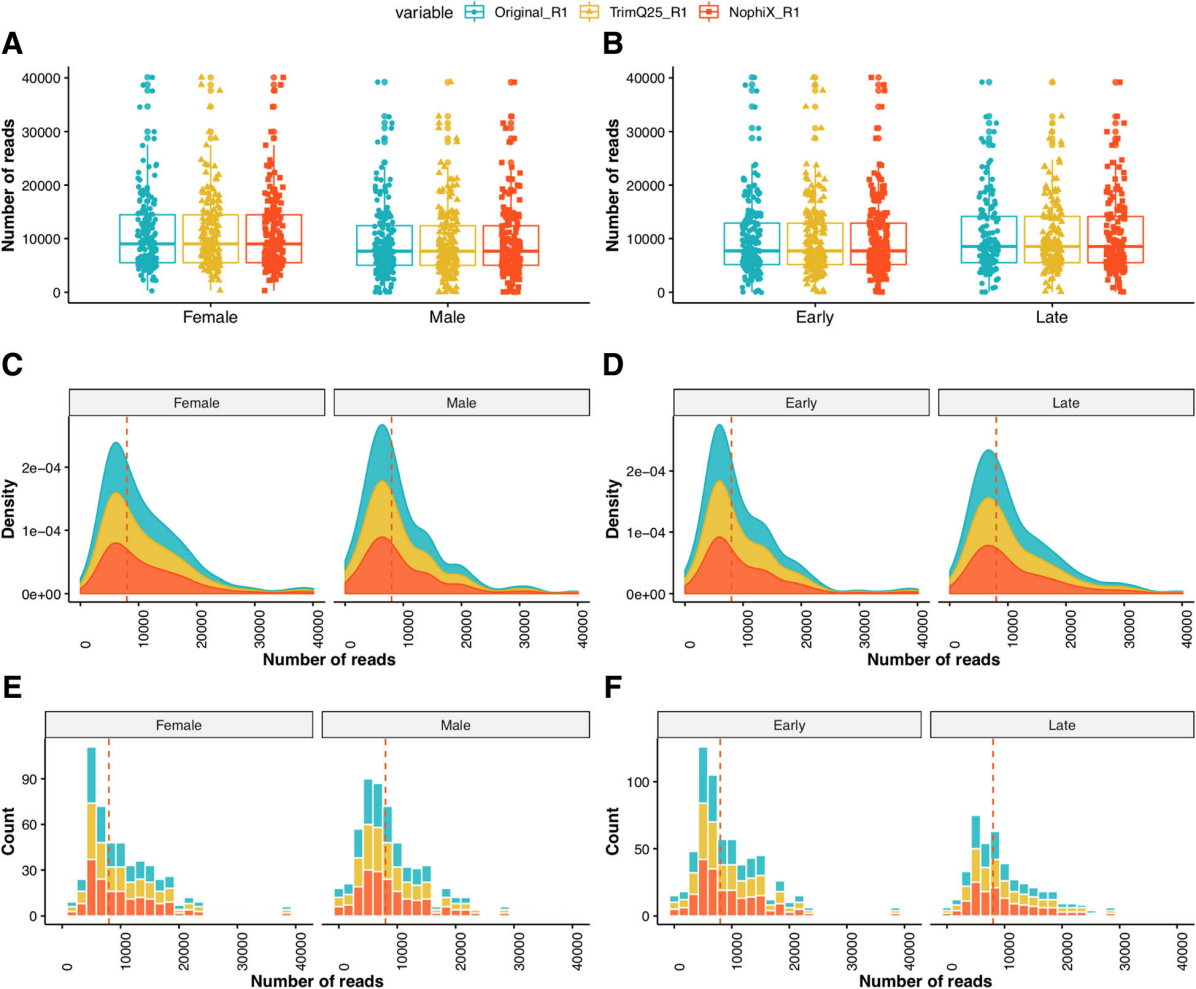
Distribution of pre-processed reads. The figure displays jittered boxplots (**a**, **b**), stacked density plots (**c**, **d**), and stacked histograms (**e**, **f**) of the forward reads. All plots give a summary of number of reads split by experimental variables; sex (male and female: **a**, **c**, **e**) and time (early and late: **b**, **d**, **f**). The legend on top of the figure shows the QC variables where Original_R1 indicates the forward reads before preprocessing, TrimQ25_R1 shows the forward after trimming at 25 Phred score, and NophiX_R2 shows the reverse reads after removing phiX contamination. Adding jitter on top of the boxplots made the variables more insightful. The line that divides the box plots into two parts and the dotted line on density plots and histograms represents the median of the data

Pre-processing results were automatically summarized into a web-based progress report 2 (Additional file 3). The whole dataset contained 3,634,461 paired-end reads. The original FastQC results showed a minimum Phred score (Q) near 10 and trimming poor quality reads at the default settings (Q = 25) and removal of phiX contaminations resulted into high quality reads (Fig. 3). Distribution of changes was visualized using boxplots, density plots and histogram plots (Fig. 4). The difference between the original and pre-processed reads was very small, barely visible in the distribution plots. Only 2692 (0.07%) poor-quality reads were identified in each forward and reverse read (Table 2) indicating that over 99.9% of the reads qualified for downstream analysis.

**Table 2:** Descriptive statistics of the pre-processed reads and total count from all samples

Statistics	Original_R1	TrimQ25_R1	NophiX_R1	Original_R2	TrimQ25_R2	NophiX_R2
Min.	14	14	14	14	14	14
1st Qu.	5368	5365	5365	5368	5365	5365
Median	8001	7996	7996	8001	7996	7996
Mean	10,096	10,089	10,088	10,096	10,089	10,088
3rd Qu.	13,636	13,630	13,630	13,636	13,630	13,630
Max.	40,113	40,077	40,077	40,113	40,077	40,077
Total reads	3,634,461	3,631,940	3,631,769	3,634,461	3,631,940	3,631,769

### Sequence processing and quality control

**Fig. 5:**
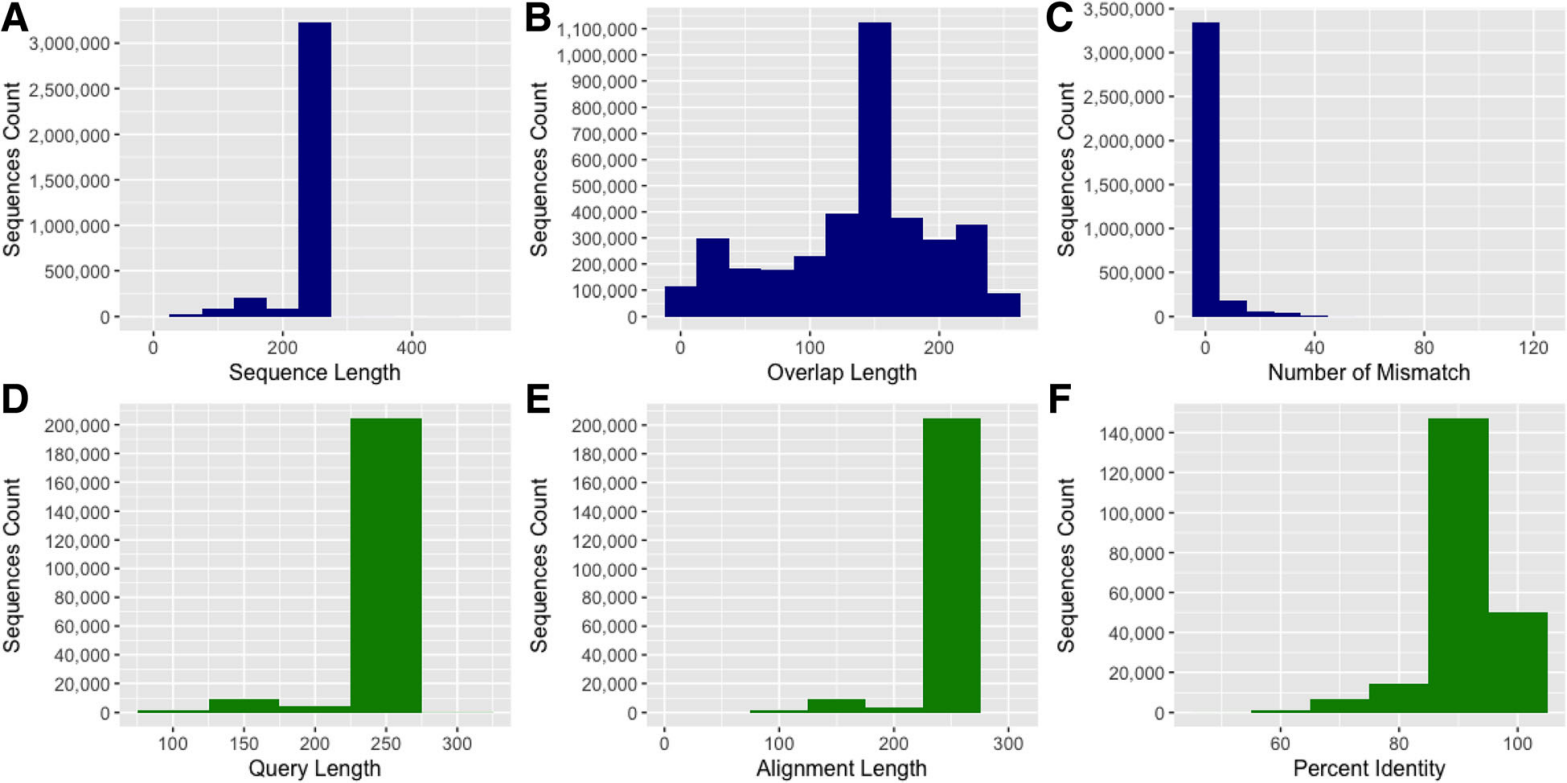
Features of the assembled and aligned sequences. Merging the forward and reverse reads resulted in sequences with 250 nucleotides (**a**). The 250-nucleotide sequence length is perfectly in-line with the targeted V4 region of the 16S rRNA gene. Most of the overlap fragments were 150 nucleotides long (**b**) and had zero mismatches (**c**). The query length (**d**) and alignment length (**e**) showed a high percent identity at 90 and 100% (**f**)

The sequence processing and taxonomy assignment results were automatically summarized into a web-based progress report 3 (Additional file 4). This process involved merging 3,631,769 high-quality read pairs to form much longer sequences that were then screened based on their length. Merging the forward and reverse reads resulted in sequences with 250 nucleotides (Fig. 5a). The 250-nucleotide sequence length is perfectly in-line with the targeted V4 region of the 16S rRNA gene. Most of the overlap fragments were 150 nucleotide long (Fig. 5b) and had mostly zero mismatches (Fig. 5c). Representative sequences (non-redundant) were then searched against SILVA rRNA reference alignments [13] to find the closest 16S rRNA gene candidates for downstream analysis. The query length (Fig. 5d) and alignment length (Fig. 5e) showed a high percent identity mostly around 90 and 100% identity (Fig. 5f). Post-alignment quality control which involved removing poor alignments and chimeric sequences yielded 2,934,726 clean sequences for downstream analysis.

### Sequence classification

**Fig. 6:**
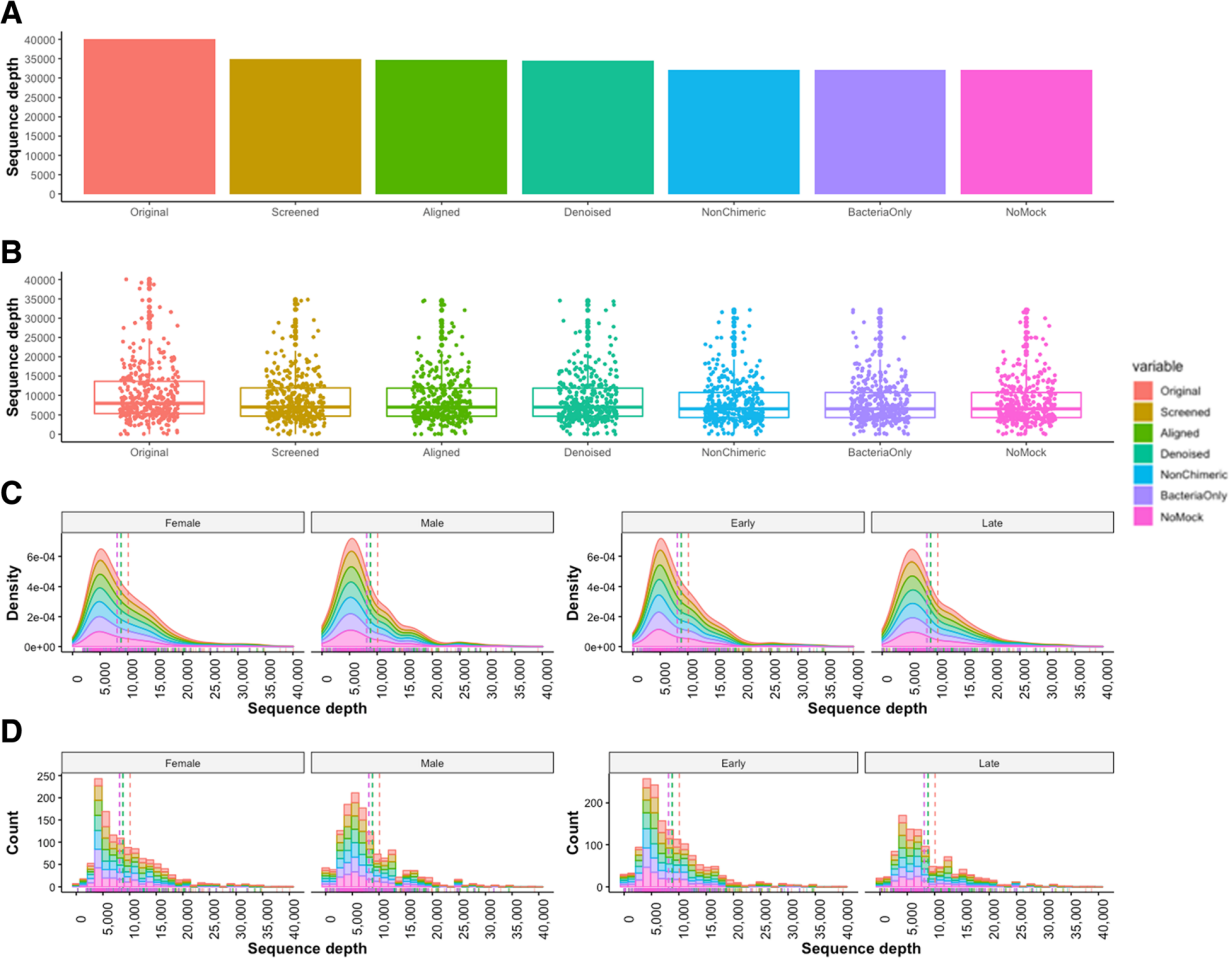
Distribution of assembled sequences after quality control. The bar plots **a** show the maximum values in each variable without much details. The jitter boxplots **b** clearly added more insights, showing the distribution, midpoint and outliers. The stacked density plots (**c**) and the stacked histograms (**d**) show the skewness of the sequence depth. Histograms separated the differences better than the other plots. Dotted lines indicate mean values of the density plots and histograms and marginal rugs are at the bottom. A slight shift of the mean line to the left is probably due to the removal of poorly aligned sequences at the denoising step. Legend key: Screened = sequences screened by length (default: min = 100, max = 300), Aligned = sequences aligned to a reference (default = SILVA alignments), Denoised = good alignments, only 1 mismatch per 100 nucleotides, NonChimeric = non chimeric sequences, BacteriaOnly = bacterial sequences only, NoMock = sequences after removing mock community

All 2,934,726 non-chimeric sequences were searched against Mothur-formatted SILVA-bacterial classifiers at 80% identity using a k-nearest neighbor consensus and Wang approach as described [20, 21]. The error rate estimated after removing any remaining non-bacterial sequences was 0.00047 (0.047%). Removal of mock community finalizes sequence processing and quality control. Tabular and graphical representation showed a slight alteration of the number of processed sequences (Table 3, Fig. 6).

**Table 3:** Descriptive statistics of processed sequences

Statistics	Original	Screened	Aligned	Denoised	NonChimeric	BacteriaOnly	NoMock
Minimum	14	8	6	6	6	6	6
1st Quantile	5365	4689	4658	4658	4336	4328	4328
Median	7996	7052	7012	7012	6590	6578	6578
Mean	10,088	8819	8767	8767	8119	8113	8113
3rd Quantile	13,630	11,937	11,870	11,870	10,740	10,733	10,733
Maximum	40,077	34,866	34,590	34,590	32,161	32,156	32,156
Total reads	3,631,769	3,174,898	3,156,040	3,156,040	2,922,704	2,920,782	2,920,782

### OTU clustering and taxonomy assignment

OTU and taxonomy results including preliminary analysis were automatically summarized into a web-based progress report 4 (Additional file 5). Clustering of 2,920,782 clean sequences into OTUs and assigning taxonomy names was done using a combination of phylotype, OTU-based and phylogeny methods as described in mothur platform [32, 33]. Taxonomy assignment in OTU-based method is by default optimized using opticlust algorithm [34]. This algorithm yielded high-quality results with high precision and low FDR ≤ 0.002 (Table 4).

**Table 4:** Statistical parameters calculated in OTU-based approach

Parameter	Value
Cutoff	0.2
Sensitivity	0.998
Specificity	0.999
PPV	0.998
NPV	0.999
Accuracy	0.999
MCC	0.997
F1 score	0.998

### OTU abundance and preliminary analysis

**Fig. 7:**
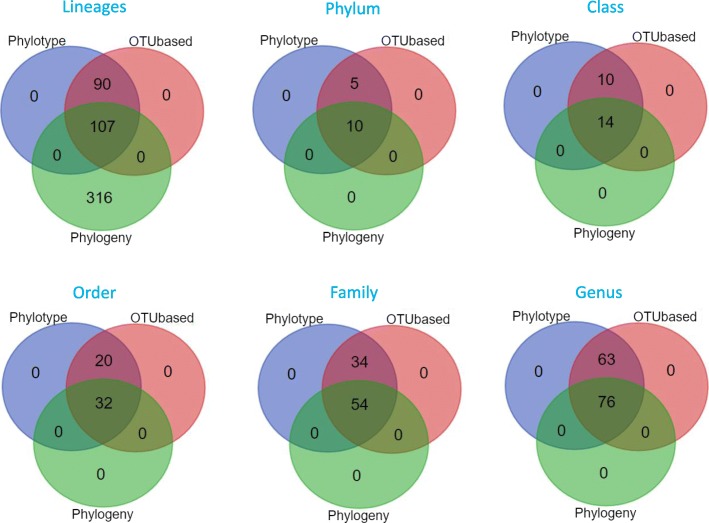
Venn diagram and taxon term representation. Visual representation of taxon terms highlighted the most abundant taxon based on the frequency of being assigned to an OTU or tree nodes. *Muribaculaceae* was the most frequently assigned family and *Muribaculaceae_ge* was the dominating genus assigned to most sequences

The phylotype method yielded 197 OTUs at genus level while 11,257 OTU clusters were generated by the OTU-based method at 97% identity. The phylogeny method generated 58,929 tree nodes which were taxonomically classified at 97% identity. As part of reviewing the intermediate results, we compared the taxonomy results across the three classification methods. A high redundancy rate was revealed where different sequences were assigned to same lineages at different percent identity, ranging from 97 to 100%, and had significantly inflated the number of OTUs, particularly in the OTU-based and phylogeny methods. We used the interactive Venn diagram viewer [35, 36] to show all possible logical relationships between the three classification methods. Briefly, the list of lineages or the taxa names was uploaded as input. The output was a tabulated textual output indicating the taxonomy lineages or taxon names that were in each intersection or unique to a specific method. Additionally, a graphical output showing the number of elements in each method in the form of symmetric Venn diagrams was generated (Table 5, Fig. 7).

**Table 5:** OTU abundance observed in three methods

Method	Lineages	Unique lineages	Unique terms		Unique taxon			
				Phylum	Class	Order	Family	Genus
Phylotype	197	197	197	15	24	52	88	139
OTU-based	11,257	197	197	15	24	52	88	139
Phylogeny	58,929	423	162	10	14	32	54	76

### Alpha diversity analysis

#### Species accumulation

**Fig. 8:**
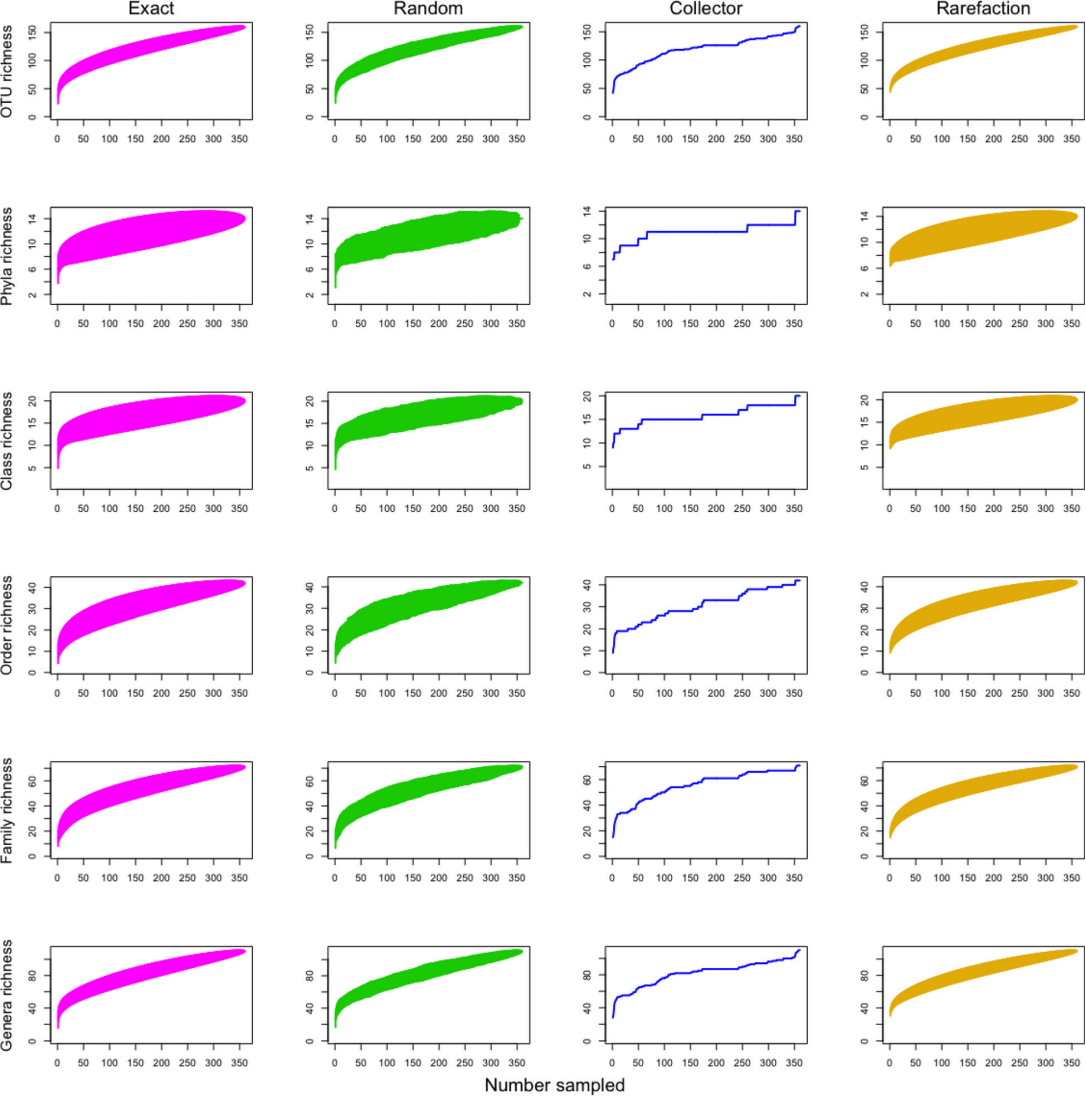
Species accumulation curves. Four methods were compared including exact (magenta), random (green), rarefaction (orange) and collector (blue). The standard deviation (except in collector curves) is indicated by the vertical lines which are highly condensed due to the large dataset (360 samples)

The number of new species added as a function of sites sampling effort was determined using four different accumulator functions as described in the vegan package [31], i.e. exact, random, collector and rarefaction (Fig. 8). Typically, the exact, random, and rarefaction methods calculate standard error bars which can guide investigators to determine which one to choose. Additionally, we used the iNEXT package [37], which enabled us to demonstrate the plotting of rarefaction and extrapolation of species diversity based on sample-size and sample coverage (see details in Additional file 5).

#### Species richness and diversity

**Fig. 9:**
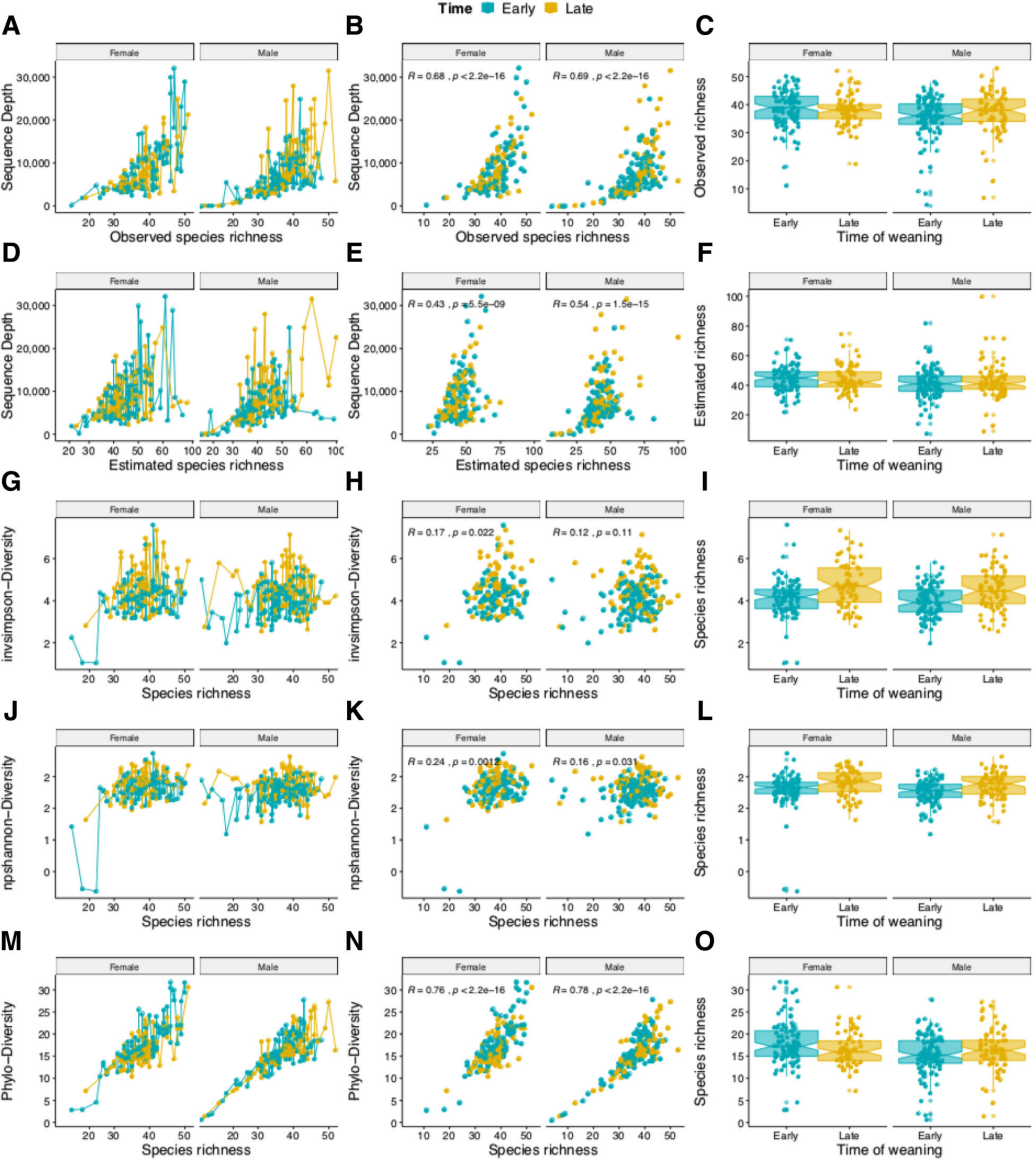
Species richness and diversity. The species richness and diversity values are displayed using line plots (left panel), the scatter diagrams with correlation coefficients (middle panel), and the jittered notched boxplots (right panel). The observed species richness (**a**, **b**, **c**) and the estimated species richness (**d**, **e**, **f**) show similar pattern. The inverse Simpson diversity index (**g**, **h**, **i**) and the Shannon diversity index (**j**, **k**, **l**) are plotted against the species richness. Both indices show similar pattern in estimating number of observed species in a sample. The Phylogenetic diversity index (**m**, **n**, **o**) shows a better direct proportion with the species richness. The figure clearly reveals the differences observed in species abundances when comparing the main experimental variables including sex (orange) and time post weaning (green)

Estimated and observed species richness were determined using Chao and Sobs calculators, respectively (Fig. 9). Three diversity indices including inverse Simpson, Shannon, and phylo-diversity were used to account for the abundance and evenness of species present in the samples.

### Beta diversity analysis

#### Clustering and ordination projections

**Fig. 10:**
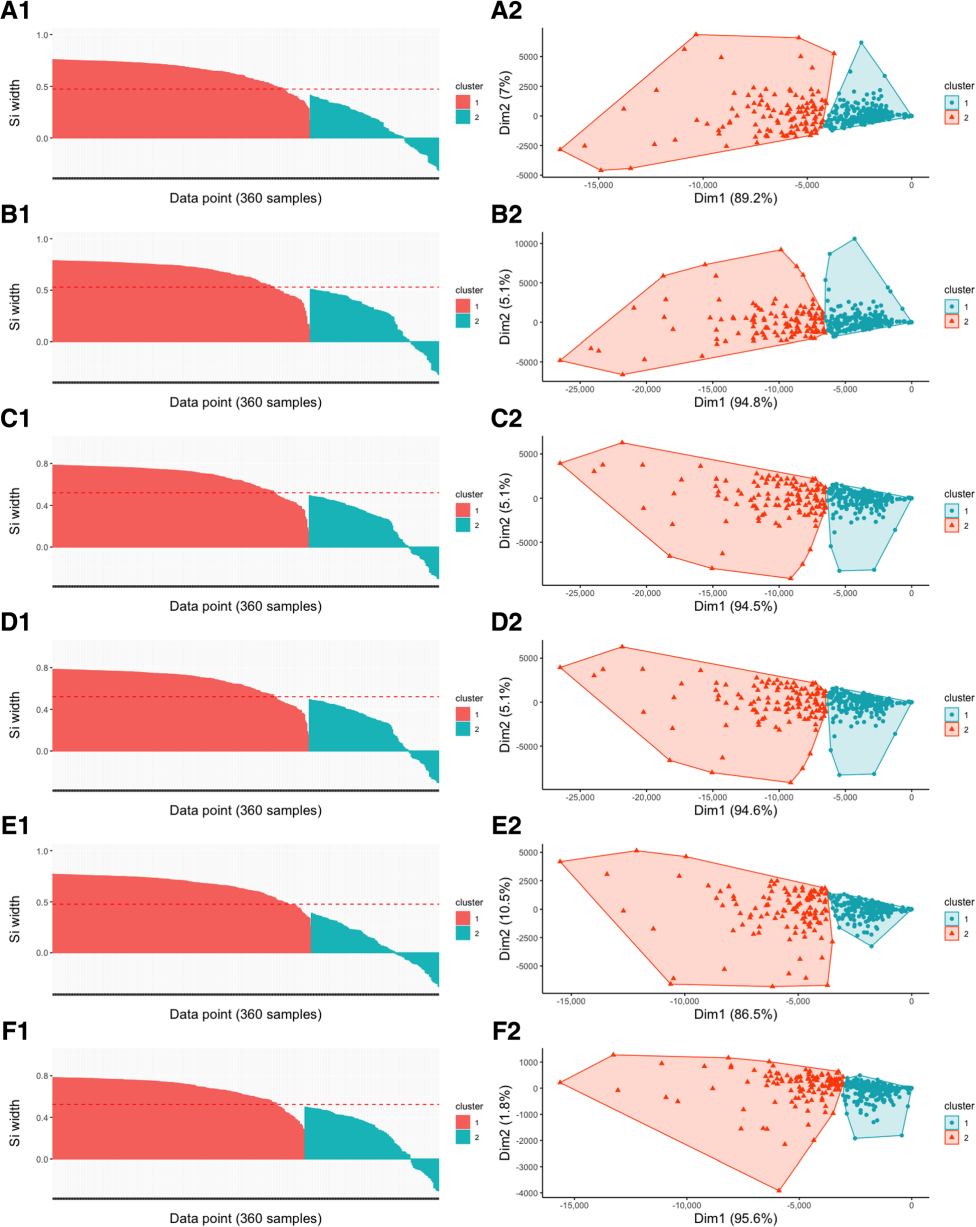
PAM clustering, Silhouette analysis and visualization of clusters. The best number of clusters obtained by PAM clustering method using scaled abundance data for the OTUs (**A1**), phylum (**B1**), class (**C1**), order (**D1**), family (**E1**), and genus (**F1**). Silhouette analysis validates the consistency within PAM clusters where scores with a large Si width (almost 1) indicates high-quality clustering, a small Si width (around 0) means that the observation lies between two clusters while negative values are outliers. The right panel shows the exploratory ordination of the samples onto two axes (Dim1 vs Dim2) based on the abundance of OTUs (**A2**), phylum (**B2**), class (**C2**), order (**D2**), family (**E2**), and genus (**F2**). Similar samples are close to each other, and dissimilar samples are farther from each other

The difference in microbial community composition across the groups was measured using the raw abundance data and the Bray-Curtis (dis)similarity coefficients. Clustering and ordination projection methods including partition around medoids (PAM) [38], principal component analysis (PCA), principal coordinate analysis (PCoA) and non-metric multidimensional scaling (NMDS) showed similar grouping (Additional file 5). A few components explained the variability of dissimilarity coefficients across the samples in all methods. Silhouette [39] graphical representation validated the consistency within PAM clusters (Fig. 10). Scree plots and data loadings were used to show components that best explained the variation in PCA and PCoA, respectively. Shepard or stress plot confirmed linearity between the original and reduced dimensions in NMDS (see details in Additional file 5).

### Phylogenetic relationship of samples

**Fig. 11:**
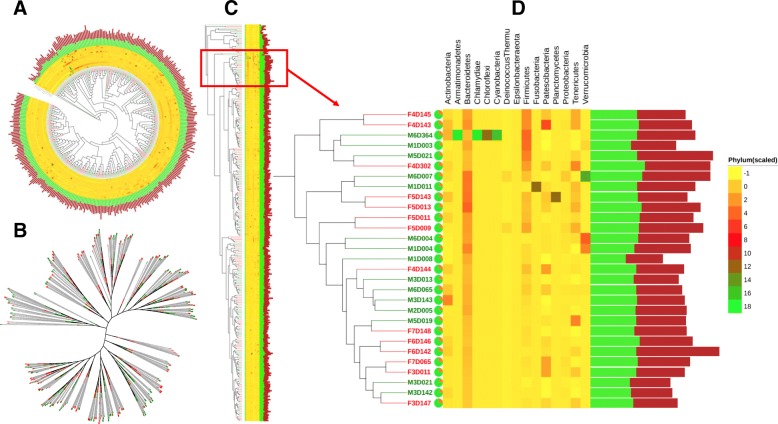
Phylogenetic relationship and annotation of samples grouped by sex variable. The circular phylograms (**a**), unrooted cladogram (**b**), and the rectangular phylograms (**c**) display the relationships of the 360 samples used in the case study. Female (red) and male (blue) linked with sequence counts showing the proportion of the number of classified (green) and unclassified (red) displayed on a pie chart followed by the phyla abundance (heatmap) and species richness bar chart showing the observed (green) and estimated (maroon) richness. A portion of the tree (**d**) is enlarged to show some details

Bray-Curtis-based Newick tree was uploaded into iTOL (Interactive Tree Of Life) viewer [24] to interactively display unrooted, circular and regular cladograms or phylograms (Fig. 11). Annotation of circular and regular cladograms with various datasets including species richness, diversity indices and relative abundances at phylum-level enabled us to see the diversity across samples.

## Discussion

We developed iMAP, a CLI-based pipeline that streamlines different functionalities from published tools, to collectively unleash the hidden biological knowledge from marker-based microbiome data. The development of this pipeline is guided by the need for a tool that is efficiently executed by a novice user to investigate bacterial communities represented in diverse samples. The iMAP pipeline is integrated with custom functions that generate reports progressively to facilitate RAYG (review-as-you-go), a new approach associated with the pipeline to enable the investigators to review the intermediate output graphically and correct any obvious errors that may lead to wrong or misleading conclusions.

The iMAP pipeline supports a wide range of fundamental analyses for profiling microbial communities present in an environmental sample. Currently, the pipeline operates on demultiplexed data generated by the Illumina platform and performs metadata profiling, tunable quality filtering, sequence processing, and classification before clustering the representative sequences into OTUs and conserved taxonomy assignment. The idea of implementing RAYG approach at every major step gives the investigators an opportunity of taking care of issues that could result in spurious OTUs and misleading conclusions.

The output generated from the major analysis steps described in the workflow (Fig. 1) is further transformed to simplify exploratory data analysis. We know that some of the intermediate output can be very large to explore but, in such a situation, iMAP uses custom scripts to extract vital information, then transform it for applying in diverse visualization modules. Reproducibility of the iMAP pipeline is ensured by including in the repository the code used for bioinformatics analysis and some custom R-based scripts for generating publication-ready images commonly reported in microbiome-related manuscripts. Users may want to explore the bioinformatics analysis results using methods of their choice or may modify provided scripts to best describe the different types of data being analyzed. Examples of publication-ready images are presented in each progress report (Additional files 2, 3, 4 and 5). Choice of which type of visualization to use is entirely user-dependent and could also depend on how much detail is required.

Post-classification annotation emphasized in this manuscript aim at making the results more accurate. A good example is the number of OTUs identified using different methods where a large was due to the high redundancy rate. Frequently, observed OTU abundance is published unfiltered. While this is not surprising, it can result in misleading conclusions. The fact that there could be some species that contribute disproportionately to the community, it would be sensible to stratify the analyses or performing intensive data annotation afterward. In microbiome data analysis it is normal to see exceptionally abundant species analyzed in the same way as those that are extremely rare. The situation is even worse if the high abundance values are influenced by having lots of redundant values. Adopting focused annotation is the key to achieving appropriate conclusions.

The preliminary analysis workflow included in the pipeline provides several methods for helping users in assessing diversity and statistical comparisons of the variables studied. Species accumulation and rarefaction, for example, provide the best way that allows investigators to figure out whether to continue sampling or whether the data is not enough for drawing a valid conclusion or for estimating normalized sample size for statistical comparisons. Other methods such as heatmap, PAM clustering, and phylogenetic analysis are integrated into the pipeline to find out the relationship of the samples and groups while ordination projections using multivariate statistical techniques such as PCA, PCoA, and NMDS can be used to identify factors explaining differences among microbial communities. We also provide statistical methods recommended in the mothur platform to compare the experimental variables. AMOVA, HOMOVA, and ANOSIM are among the methods that use *P*-values to determine if the observed differences are statistically significant or are by chance. The Metastats program [40] can be used for detecting differentially abundant microbial communities while the Kruskal–Wallis one-way ANOVA is commonly used to determine if there are statistically significant differences between two or more groups. The mothur-based lefse command modeled after the LEfSe program [41] is an excellent tool for biomarker discovery while the weighted and unweighted UniFrac [42] can be used to compare the samples using their phylogenetic information.

The iMAP pipeline has been successfully tested in-house using multiple datasets from our bushmeat project (Project # HDTRA1–16-1-0005). Currently, it is being used to analyze 186 bushmeat samples to characterize the spectrum of microbes present in market bushmeat in Tanzania. The analysis will integrate three groups of variables including three ecosystems (Serengeti, Ruaha, and Selous), two seasons (wet and dry) and two conditions (fresh and processed).

## Conclusions

The iMAP pipeline wraps bioinformatics and visualization tool for generating high-quality user-reviewed microbiome data analysis output. The pipeline integrates read preprocessing, quality control, sequence classification, and OTU taxonomy assignment workflows with data visualization tools to produce high-quality output and diverse visuals for better understanding of complex and multidimensional microbiome data. It also integrates functions that generate reports progressively, emphasizing on the RAYG (review-as-you-go) approach, especially for the in-between process output. Integrating the iMAP with RAYG approach enables the investigators to discover and correct any systematic errors that could lead to misleading conclusions. The multiple statistical methods incorporated in the pipeline guide the investigators to make well-informed decisions and predictions backed by data as well as generating data-driven hypotheses. We believe that users will find this tool broadly useful and adaptable to their microbiome data analysis needs.

## Availability and requirements

Project name: e.g. iMAP

Project home page: https://github.com/tmbuza/iMAP

Operating system(s): Platform independent if using the associated Docker images. Mac OS X or Linux CLI if run outside Docker images.

Programming language: Bash, R 3.5, Perl, Python.

Other requirements: Mothur v1.42 or higher, QIIME2 v2019, Java JDK, Chrome, Firefox, and Safari web browser.

License: MIT

Any restrictions to use by non-academics: None.

## Additional files

Additional file 1: Format of input files. Includes sample-metadata mapping (sheet 1), sample-read-file mapping in mothur-format (sheet2), and sample-variable mapping (sheet 3, 4 and 5). (XLSX 69 kb)

Additional file 2: Metadata profiling report generated automatically by the iMAP to provide a summary of the samples and the associated metadata. This report is the initial step in the RAYG (review-as-go) process. The report also displays the R-commands that demonstrates how to reproduce the report. The pipeline is set to automatically save the output in the “reports” folder as “report1_metadata_profiling.html”. (HTML 953 kb)

Additional file 3: Preprocessing report generated automatically by the iMAP to provide a summary of quality control of the reads. The iMAP pipeline automatically saved the output in the “reports” folder as “report2_read_preprocessing.html”. (HTML 3463 kb)

Additional file 4:  Sequence processing report generated automatically by the iMAP to provide a summary of the output.  The report was automatically saved in the “reports” folder as “report3_sequence_processing.html”. (HTML 4205 kb)

Additional file 5:  Preliminary analysis report generated automatically by the iMAP to provide a summary of conserved taxonomy assigned to OTUs and the initial analysis of OTUs and taxa data. The preliminary analysis report was automatically saved in the “reports” folder as “report4_preliminary_analysis.html”. (HTML 20379 kb)

## Data Availability

The dataset analyzed during the current study as a case study is publicly available at http://www.mothur.org/MiSeqDevelopmentData/StabilityNoMetaG.tar. The data supporting the results reported in this manuscript is included within the article and its additional files. The auto-generate progress reports (Additional files 2, 3, 4 and 5) are in HTML format and can be viewed using any preferred browser such as Chrome, Safari, Internet Explorer and Firefox. The iMAP repository which also includes the entire code and other requirements can be downloaded from https://github.com/tmbuza/iMAP. The guidelines for implementing iMAP and related updates, are available at: https://github.com/tmbuza/iMAP/blob/master/README.md. For further inquiries, please contact the corresponding author at tmbuza@microbiome-bioinfo.com.

## Abbreviations

AMOVA: Analysis of Molecular Variance
ANOSIM: Analysis of Similarity
CLI: Command-Line Interface
FDR: False Discovery Rate
GUI: Graphical User Interface
HOMOVA: Homogeneity of Variance
IDE: Integrated Development Environment
iMAP: Integrated Microbiome Analysis Pipeline
MCC: Matthews Correlation Coefficient
NPV: Negative Predictive Value
OTU: Operational Taxonomic Unit
PAM: Partitioning Around Medoids
PCA: Principal Component Analysis
PCoA: Principal Coordinates Analysis
PPV: Positive Predictive Value
PSU-ICS: Penn State University-Institute for CyberScience
RAYG: Review-As-You-Go
UPGMA: Unweighted Pair Group Method with Arithmetic Mean
