# Supplementary material for: iMAP: an integrated bioinformatics and visualization pipeline for microbiome data analysis
Source: BMC Bioinformatics. 2019 Jul 3;20:374. doi: 10.1186/s12859-019-2965-4 (PMC6610863; doi:10.1186/s12859-019-2965-4)
Supplement: Supplementary file 2 — Metadata profiling report generated automatically by the iMAP to provide a summary of the samples and the associated metadata. This report is the initial step in the RAYG (review-as-go) process. The report also displays the R-commands that demonstrates how to reproduce the report. The pipeline is set to automatically save the output in the “reports” folder as “report1_metadata_profiling.html”. (HTML 953 kb) [file 12859_2019_2965_MOESM2_ESM.html]

Progress report 1


# Progress report 1

### Metadata profiling

#### Updated: 2019-05-15 11:55:57

## Preamble

- Load Metadata
  - Inspect the metadata column names.
  - Remove number sign at the name of column one.
  - Note that Var1 starts at index 7.
  - Select important variables for downstream analysis.
  - Define each categoric variable as factor.

```
# Load samplemetadata
samplemetadata <- readr::read_table2(file="~/OneDrive/CaseStudy/iMAPpipeline_rev2/iMAPdemo/iMAP/resources/metadata/samplemetadata.tsv", col_names = T)
colnames(samplemetadata)[1] <- "SampleID"

# View column names
names(samplemetadata)
```

```
 [1] "SampleID"              "BarcodeSequence"      
 [3] "ForwardPrimerSequence" "ReversePrimerSequence"
 [5] "ForwardRead"           "ReverseRead"          
 [7] "Var1"                  "Var2"                 
 [9] "Var3"                  "Var4"                 
[11] "Description"
```

```
# Define categoric variables like
samplemetadata$SampleID <- as.factor(samplemetadata$SampleID)
samplemetadata$Var1 <- as.factor(samplemetadata$Var1)
samplemetadata$Var2 <- as.factor(samplemetadata$Var2)
samplemetadata$Var3 <- as.factor(samplemetadata$Var3)
samplemetadata$Var4 <- as.factor(samplemetadata$Var4)

# Add more variables as needed
```

### Status of sample metadata

```
library(dplyr)
library(knitr)

# show data
metadatastatus %>% knitr::kable()
```

| variable | q\_zeros | p\_zeros | q\_na | p\_na | q\_inf | p\_inf | type | unique |
| --- | --- | --- | --- | --- | --- | --- | --- | --- |
| SampleID | 0 | 0 | 0 | 0 | 0 | 0 | factor | 8 |
| BarcodeSequence | 0 | 0 | 8 | 100 | 0 | 0 | logical | 0 |
| ForwardPrimerSequence | 0 | 0 | 8 | 100 | 0 | 0 | logical | 0 |
| ReversePrimerSequence | 0 | 0 | 8 | 100 | 0 | 0 | logical | 0 |
| ForwardRead | 0 | 0 | 0 | 0 | 0 | 0 | character | 8 |
| ReverseRead | 0 | 0 | 0 | 0 | 0 | 0 | character | 8 |
| Var1 | 0 | 0 | 0 | 0 | 0 | 0 | factor | 2 |
| Var2 | 0 | 0 | 0 | 0 | 0 | 0 | factor | 2 |
| Var3 | 0 | 0 | 0 | 0 | 0 | 0 | factor | 4 |
| Var4 | 0 | 0 | 0 | 0 | 0 | 0 | factor | 4 |
| Description | 0 | 0 | 0 | 0 | 0 | 0 | character | 1 |

Key: **q\_zeros**: quantity of missing data; **p\_zeros**: percentage of missing data, **q\_na**: quantity of NA; **p\_na**: percentage of NA, **q\_inf**: quantity of infinite values; **p\_inf**: percentage of infinity values, **type**: factor, character, integer or numeric; **unique**: levels of the variable.

### Selected variables for downsteam analysis

> This is important for mapping to output from dowmstream analysis

```
library(dplyr)
library(knitr)

# show variable status
varstatus %>% knitr::kable()
```

| variable | q\_zeros | p\_zeros | q\_na | p\_na | q\_inf | p\_inf | type | unique |
| --- | --- | --- | --- | --- | --- | --- | --- | --- |
| SampleID | 0 | 0 | 0 | 0 | 0 | 0 | factor | 8 |
| Var1 | 0 | 0 | 0 | 0 | 0 | 0 | factor | 2 |
| Var2 | 0 | 0 | 0 | 0 | 0 | 0 | factor | 2 |
| Var3 | 0 | 0 | 0 | 0 | 0 | 0 | factor | 4 |
| Var4 | 0 | 0 | 0 | 0 | 0 | 0 | factor | 4 |

Key: **q\_zeros**: quantity of missing data; **p\_zeros**: percentage of missing data, **q\_na**: quantity of NA; **p\_na**: percentage of NA, **q\_inf**: quantity of infinite values; **p\_inf**: percentage of infinity values, **type**: factor, character, integer or numeric; **unique**: frequency of the values.

## View variable levels (first 5 rows)

```
variables %>% head(5)  %>% knitr::kable()
```

| SampleID | Var1 | Var2 | Var3 | Var4 |
| --- | --- | --- | --- | --- |
| F3D001 | Female | Early | D001 | 1 |
| F3D006 | Female | Early | D006 | 6 |
| F3D142 | Female | Late | D142 | 142 |
| F3D143 | Female | Late | D143 | 143 |
| M1D001 | Male | Early | D001 | 1 |

  


---

## Frequency of experimental variables

```
library(ggplot2)

# Var1
var1histplot <- ggpubr::gghistogram(variables, x = "Var1", stat="count", fill = "#ff0000") + 
  labs(x = "Variable 1", y = "Sample count") 
  
# Var2
var2histplot <- ggpubr::gghistogram(variables, x = "Var2", stat="count", fill = "#00ff00") + 
  labs(x = "Variable 2", y = "Sample count") 

# Var3
var3histplot <- ggpubr::gghistogram(variables, x = "Var3", stat="count", fill = "#0000ff") + 
  theme(axis.text.x=element_text(angle = 0, hjust = 0))+ scale_y_continuous(labels=scaleFUN) +
  labs(x = "Variable 3", y = "Sample count") 

ggpubr::ggarrange(var1histplot,var2histplot, var3histplot, ncol = 1, nrow = 3, align = "hv", labels = c("AUTO"))
```

### Profiling metadata used in the iMAP casestudy

> Using the Casestudy metadata located in this path: iMAP/resources/metadata/samplemetadata\_casestudy.tsv

```
library("dplyr")
library("knitr")

# Load samplemetadata

samplemetadata2 <- readr::read_table2(file="~/OneDrive/CaseStudy/iMAPpipeline_rev2/iMAPdemo/iMAP/resources/metadata/samplemetadata_casestudy.tsv", col_names = T)
samplemetadata2 <- samplemetadata2[, -7]
colnames(samplemetadata2)[1] <- "SampleID"

# View column names
names(samplemetadata2)
```

```
 [1] "SampleID"              "BarcodeSequence"      
 [3] "ForwardPrimerSequence" "ReversePrimerSequence"
 [5] "ForwardRead"           "ReverseRead"          
 [7] "Sex"                   "Time"                 
 [9] "DayID"                 "DPW"                  
[11] "Description"
```

```
# Define categoric variables like
samplemetadata2$SampleID <- as.factor(samplemetadata2$SampleID)
samplemetadata2$Sex <- as.factor(samplemetadata2$Sex)
samplemetadata2$Time <- as.factor(samplemetadata2$Time)
samplemetadata2$DayID <- as.factor(samplemetadata2$DayID)
samplemetadata2$DPW <- as.factor(samplemetadata2$DPW)

# Add more variables as needed

variables2 <- samplemetadata2  %>% dplyr::select("SampleID", "Sex", "Time", "DayID", "DPW" )
```

## Status of custom metadata

```
library(dplyr)
library(knitr)

# show data
varstatus2 %>% knitr::kable()
```

| variable | q\_zeros | p\_zeros | q\_na | p\_na | q\_inf | p\_inf | type | unique |
| --- | --- | --- | --- | --- | --- | --- | --- | --- |
| SampleID | 0 | 0.00 | 0 | 0 | 0 | 0 | factor | 360 |
| Sex | 0 | 0.00 | 0 | 0 | 0 | 0 | factor | 2 |
| Time | 0 | 0.00 | 0 | 0 | 0 | 0 | factor | 2 |
| DayID | 0 | 0.00 | 0 | 0 | 0 | 0 | factor | 35 |
| DPW | 12 | 3.33 | 0 | 0 | 0 | 0 | factor | 35 |

Key: **q\_zeros**: quantity of missing data; **p\_zeros**: percentage of missing data, **q\_na**: quantity of NA; **p\_na**: percentage of NA, **q\_inf**: quantity of infinite values; **p\_inf**: percentage of infinity values, **type**: factor, character, integer or numeric; **unique**: levels of the variable.

When reviewing metadata status you may notice that numeric variables are confused with numeric data. For example, variable **DPW** (days post weaning) on Table x shows 12 missing values (3.33%) which is incorrect. As investigators we know that day 1 was coded zero (0) (12 zeros for 12 mouse) which in descriptive statistics is interpreted as missing data. To correct this we re-coded the samples with unique identifies shown as DayID to distinguish them from integers. However, depending of experiment this kind of variables need to be converted to character during analysis.

## Frequency of experimental variables in the Demo dataset

```
library(ggplot2)
#sort by dpw (descending)

sexhistplot <- ggpubr::gghistogram(variables2, x = "Sex", stat="count", fill = "#ff0000") + 
  labs(x = "Sex of animal", title = "Sex variable")

timehistplot <- ggpubr::gghistogram(variables2, x = "Time", stat="count", fill = "#00ff00") + 
  labs(x = "Time of Weaning", title = "Time variable")

dpwhistplot <- ggpubr::gghistogram(variables2, x = "DayID", stat="count", fill = "#0000ff") +
  theme(axis.text.x=element_text(angle = 90, hjust = 0))+ scale_y_continuous(labels=scaleFUN) + 
  labs(x = "Day Code", title = "Sex variable")

ggpubr::ggarrange(sexhistplot,timehistplot, dpwhistplot, ncol = 1, nrow = 3,  common.legend = TRUE, legend = "top", align = "hv", labels = c(""))
```

```
save(samplemetadata, samplemetadata2, variables, variables2, file = "~/OneDrive/CaseStudy/iMAPpipeline_rev2/iMAPdemo/iMAP/code/Rmd/metadatavars.RData")
save(samplemetadata, samplemetadata2, variables, variables2, file = "~/GitHub/MAY2019/iMAP/code/Rmd/metadatavars.RData")
```

  


---

## Posible questions

### Metadata profiling

- QN1: Are there samples to be removed from the analysis based on their count?
- QN2: Are there enough variables for downstream diversity and statistical analysis?
- QN3: …….?
- QN4: …….?
- QN5: …….?

## Summary of packages used in the analysis

```
sessionInfo()
```

```
R version 3.5.2 (2018-12-20)
Platform: x86_64-apple-darwin15.6.0 (64-bit)
Running under: macOS Mojave 10.14.4

Matrix products: default
BLAS: /Library/Frameworks/R.framework/Versions/3.5/Resources/lib/libRblas.0.dylib
LAPACK: /Library/Frameworks/R.framework/Versions/3.5/Resources/lib/libRlapack.dylib

locale:
[1] en_US.UTF-8/en_US.UTF-8/en_US.UTF-8/C/en_US.UTF-8/en_US.UTF-8

attached base packages:
[1] stats     graphics  grDevices utils     datasets  methods   base     

other attached packages:
[1] ggplot2_3.1.0 knitr_1.22    dplyr_0.8.0.1

loaded via a namespace (and not attached):
  [1] nlme_3.1-137        bitops_1.0-6        phyloseq_1.26.1    
  [4] RColorBrewer_1.1-2  tools_3.5.2         backports_1.1.3    
  [7] R6_2.4.0            vegan_2.5-4         KernSmooth_2.23-15 
 [10] rpart_4.1-13        Hmisc_4.2-0         lazyeval_0.2.1     
 [13] BiocGenerics_0.28.0 mgcv_1.8-27         colorspace_1.4-1   
 [16] permute_0.9-4       ade4_1.7-13         nnet_7.3-12        
 [19] withr_2.1.2         tidyselect_0.2.5    gridExtra_2.3      
 [22] moments_0.14        compiler_3.5.2      microbiome_1.4.2   
 [25] Biobase_2.42.0      htmlTable_1.13.1    iNEXT_2.0.19       
 [28] xml2_1.2.0          NLP_0.2-0           labeling_0.3       
 [31] entropy_1.2.1       slam_0.1-45         caTools_1.17.1.1   
 [34] scales_1.0.0        checkmate_1.9.1     tm_0.7-6           
 [37] readr_1.3.1         stringr_1.4.0       digest_0.6.18      
 [40] foreign_0.8-71      rmarkdown_1.12      XVector_0.22.0     
 [43] base64enc_0.1-3     pkgconfig_2.0.2     htmltools_0.3.6    
 [46] highr_0.8           htmlwidgets_1.3     rlang_0.3.4        
 [49] rstudioapi_0.10     jsonlite_1.6        gtools_3.8.1       
 [52] acepack_1.4.1       magrittr_1.5        Formula_1.2-3      
 [55] biomformat_1.10.1   Matrix_1.2-15       Rcpp_1.0.1         
 [58] munsell_0.5.0       S4Vectors_0.20.1    Rhdf5lib_1.4.3     
 [61] ape_5.2             stringi_1.4.3       yaml_2.2.0         
 [64] MASS_7.3-51.1       zlibbioc_1.28.0     rhdf5_2.26.2       
 [67] gplots_3.0.1.1      plyr_1.8.4          grid_3.5.2         
 [70] parallel_3.5.2      gdata_2.18.0        crayon_1.3.4       
 [73] lattice_0.20-38     cowplot_0.9.4       Biostrings_2.50.2  
 [76] splines_3.5.2       pander_0.6.3        multtest_2.38.0    
 [79] hms_0.4.2           funModeling_1.7     pillar_1.3.1       
 [82] igraph_1.2.4        ggpubr_0.2          reshape2_1.4.3     
 [85] codetools_0.2-16    stats4_3.5.2        glue_1.3.1         
 [88] evaluate_0.13       latticeExtra_0.6-28 data.table_1.12.0  
 [91] foreach_1.4.4       gtable_0.2.0        purrr_0.3.2        
 [94] tidyr_0.8.3         assertthat_0.2.1    xfun_0.6           
 [97] survival_2.43-3     tibble_2.1.1        iterators_1.0.10   
[100] IRanges_2.16.0      cluster_2.0.7-1     ROCR_1.0-7
```
