# Supplementary material for: iMAP: an integrated bioinformatics and visualization pipeline for microbiome data analysis
Source: BMC Bioinformatics. 2019 Jul 3;20:374. doi: 10.1186/s12859-019-2965-4 (PMC6610863; doi:10.1186/s12859-019-2965-4)
Supplement: Supplementary file 3 — Preprocessing report generated automatically by the iMAP to provide a summary of quality control of the reads. The iMAP pipeline automatically saved the output in the “reports” folder as “report2_read_preprocessing.html”. (HTML 3463 kb) [file 12859_2019_2965_MOESM3_ESM.html]

Progress report 2


# Progress report 2

### Pre-processing of paired-reads

#### Updated: 2019-05-15 11:53:37

s

## Read count

---

```
Number of forward reads before QC (Original_R1):     3634461 ( 100 % )
```

```
Number of reverse reads before QC (Original_R2):     3634461 ( 100 % )
```

```
Total Number of reads before QC:     7268922
```

## Read length

---

Figure x: Density and histograms plots showing forward and reverse read length. Dotted line indicates mean value for the specified variable

# Distribution of raw reads

## Barplots

---

**Grouped by Sex variable**

Figure x: Barplots of pre-processed reads grouped by sex variable.

**Grouped by Time variable**

Figure x: Barplots of pre-processed reads grouped by time variable

**Grouped by days-post-weaning (D) variable**

Figure x: Barplots of pre-processed reads grouped by time variable

# Base call quality

- In the results folder you will find a folder named *multiqc* which contains data for HTML display. Right-click-open with your favorite browser
- Using CLI: Alternatively, open the HTML file on command line by running the following commands

## Original base call quality (qc0)

---

```
open ./results/multiqc/qc0/multiqc_report.html
open ./results/multiqc/qc0/R1/multiqc_report.html
open ./results/multiqc/qc0/R2/multiqc_report.html
```

Figure x: Example of report generated by running **open ./results/multiqc/qc0/multiqc\_report.html** on CLI

## Base call quality after trimming at Q = 25 (qctrim25)

---

```
open ./results/multiqc/qctrim25/multiqc_report.html
open ./results/multiqc/qctrim25/R1/multiqc_report.html
open ./results/multiqc/qctrim25/R2/multiqc_report.html
```

Figure x: Example of report generated by running **open ./results/multiqc/qced/R2/multiqc\_report.html** on CLI

## Base call quality after removing phiX

---

```
open ./results/multiqc/qced/multiqc_report.html
open ./results/multiqc/qced/R1/multiqc_report.html
open ./results/multiqc/qced/R2/multiqc_report.html
```

Figure x: Example of report generated by running **open ./results/multiqc/qced/R2/multiqc\_report.html** on CLI

# Distribution of pre-processed reads

- Bar plots
- Box plots
- Density plots
- Histogram plots

## Stacked barplot

---

Figure x: Stacked barplots of pre-processed reads grouped by QC variable

## Boxplots

---

## Density plots

---

## Histograms

---

## Grouped by variable

# Summary of pre-processed reads

## Total read count

---

- Sum of reads in all samples

```
Number of forward reads before QC (Original_R1):     3634461 ( 100 % )
```

```
Number of reverse reads before QC (Original_R2):     3634461 ( 100 % )
```

```
Number of forward reads after trimming at Q=25 (TrimQ25_R1):     3631940 ( 99.93064 % )
```

```
Number of reverse reads after trimming at Q=25 (TrimQ25_R2):     3631940 ( 99.93064 % )
```

```
Number of forward reads after phiX removal (NophiX_R1):  3631769 ( 99.92593 % )
```

```
Number of reverse reads after phiX removal (NophiX_R2):  3631769 ( 99.92593 % )
```

## Descriptive statistics

---

> Shows summary of the exact number of sequences remaining at each step in 4-quantiles.

```
  Original_R1      TrimQ25_R1      NophiX_R1      Original_R2   
 Min.   :   14   Min.   :   14   Min.   :   14   Min.   :   14  
 1st Qu.: 5368   1st Qu.: 5365   1st Qu.: 5365   1st Qu.: 5368  
 Median : 8001   Median : 7996   Median : 7996   Median : 8001  
 Mean   :10096   Mean   :10089   Mean   :10088   Mean   :10096  
 3rd Qu.:13636   3rd Qu.:13630   3rd Qu.:13630   3rd Qu.:13636  
 Max.   :40113   Max.   :40077   Max.   :40077   Max.   :40113  
   TrimQ25_R2      NophiX_R2    
 Min.   :   14   Min.   :   14  
 1st Qu.: 5365   1st Qu.: 5365  
 Median : 7996   Median : 7996  
 Mean   :10089   Mean   :10088  
 3rd Qu.:13630   3rd Qu.:13630  
 Max.   :40077   Max.   :40077
```

# Posible questions

---

## At read inspection step

- QN1: Are there samples to be removed from the analysis based on read depth?
- QN2: Are there samples to be removed from the analysis based on read length?
- QN3: …….?
- QN4: …….?

## At base call quality checking step

- QN1: Are there samples to be removed from the analysis based on read quality?
- QN2: What are the minimum trimming parameters?
- QN3: Is trimming both ends necessary?
- QN4: Was phiX control used in the experiment, if so should removal of phiX contamination necessary?

## Summary of packages used in the analysis

```
R version 3.5.2 (2018-12-20)
Platform: x86_64-apple-darwin15.6.0 (64-bit)
Running under: macOS Mojave 10.14.4

Matrix products: default
BLAS: /Library/Frameworks/R.framework/Versions/3.5/Resources/lib/libRblas.0.dylib
LAPACK: /Library/Frameworks/R.framework/Versions/3.5/Resources/lib/libRlapack.dylib

locale:
[1] en_US.UTF-8/en_US.UTF-8/en_US.UTF-8/C/en_US.UTF-8/en_US.UTF-8

attached base packages:
[1] stats     graphics  grDevices utils     datasets  methods   base     

other attached packages:
[1] ggpubr_0.2    magrittr_1.5  dplyr_0.8.0.1 ggplot2_3.1.0

loaded via a namespace (and not attached):
 [1] Rcpp_1.0.1       pillar_1.3.1     compiler_3.5.2   plyr_1.8.4      
 [5] tools_3.5.2      digest_0.6.18    evaluate_0.13    tibble_2.1.1    
 [9] gtable_0.2.0     pkgconfig_2.0.2  rlang_0.3.4      yaml_2.2.0      
[13] xfun_0.6         gridExtra_2.3    withr_2.1.2      stringr_1.4.0   
[17] knitr_1.22       hms_0.4.2        grid_3.5.2       tidyselect_0.2.5
[21] cowplot_0.9.4    glue_1.3.1       R6_2.4.0         rmarkdown_1.12  
[25] purrr_0.3.2      readr_1.3.1      reshape2_1.4.3   scales_1.0.0    
[29] htmltools_0.3.6  assertthat_0.2.1 colorspace_1.4-1 labeling_0.3    
[33] stringi_1.4.3    lazyeval_0.2.1   munsell_0.5.0    crayon_1.3.4
```
