# Supplementary material for: iMAP: an integrated bioinformatics and visualization pipeline for microbiome data analysis
Source: BMC Bioinformatics. 2019 Jul 3;20:374. doi: 10.1186/s12859-019-2965-4 (PMC6610863; doi:10.1186/s12859-019-2965-4)
Supplement: Supplementary file 4 — Sequence processing report generated automatically by the iMAP to provide a summary of the output. The report was automatically saved in the “reports” folder as “report3_sequence_processing.html”. (HTML 4205 kb) [file 12859_2019_2965_MOESM4_ESM.html]

Progress report 3


# Progress report 3

### Sequence Processing and classification

#### Updated: 2019-05-15 11:50:31

## Descriptive statistics of assembled sequence

|  | SequenceLength | OverlapLength | MisMatches |
| --- | --- | --- | --- |
|  | Min. : 14 | Min. : 0.0 | Min. : 0.00 |
|  | 1st Qu.:252 | 1st Qu.:102.0 | 1st Qu.: 0.00 |
|  | Median :252 | Median :153.0 | Median : 0.00 |
|  | Mean :241 | Mean :137.7 | Mean : 1.38 |
|  | 3rd Qu.:253 | 3rd Qu.:173.0 | 3rd Qu.: 0.00 |
|  | Max. :500 | Max. :250.0 | Max. :116.00 |

## Features of assembled sequences

## Descriptive statistics of aligned sequence

|  | QueryLength | AlignmentLength | PercentIdentity |
| --- | --- | --- | --- |
|  | Min. :100.0 | Min. :-1595.0 | Min. : 50.00 |
|  | 1st Qu.:252.0 | 1st Qu.: 252.0 | 1st Qu.: 89.68 |
|  | Median :253.0 | Median : 253.0 | Median : 92.09 |
|  | Mean :246.4 | Mean : 246.5 | Mean : 91.30 |
|  | 3rd Qu.:253.0 | 3rd Qu.: 253.0 | 3rd Qu.: 94.86 |
|  | Max. :300.0 | Max. : 303.0 | Max. :100.00 |

## Features of aligned sequences

## Publication-ready figure

> Subplots of assembled (blue) and aligned (green) sequences are grouped together

## View the first 10 rows of prosecced data

| SampleID | Original | Screened | Aligned | Denoised | NonChimeric | BacteriaOnly | NoMock |
| --- | --- | --- | --- | --- | --- | --- | --- |
| F3D000 | 7786 | 6836 | 6813 | 6810 | 6374 | 6369 | 6369 |
| F3D001 | 5862 | 5026 | 5009 | 5009 | 4712 | 4705 | 4705 |
| F3D002 | 19610 | 17355 | 17277 | 15105 | 13918 | 13852 | 13852 |
| F3D003 | 6756 | 5955 | 5920 | 11068 | 10057 | 10055 | 10055 |
| F3D005 | 4444 | 3861 | 3844 | 14846 | 13869 | 13831 | 13831 |
| F3D006 | 7985 | 7048 | 7014 | 5224 | 4784 | 4784 | 4784 |
| F3D007 | 5124 | 4538 | 4510 | 2773 | 2521 | 2516 | 2516 |
| F3D008 | 5292 | 4639 | 4611 | 2763 | 2472 | 2472 | 2472 |
| F3D009 | 7065 | 6247 | 6208 | 4048 | 3596 | 3596 | 3596 |
| F3D011 | 17774 | 15226 | 15127 | 6353 | 5734 | 5732 | 5732 |

## Summary statistics of processed sequences

> Shows summary of the exact number of sequences remaining at each step in 4-quantiles.

```
    Original        Screened        Aligned         Denoised    
 Min.   :   14   Min.   :    8   Min.   :    6   Min.   :    6  
 1st Qu.: 5365   1st Qu.: 4688   1st Qu.: 4658   1st Qu.: 4656  
 Median : 7996   Median : 7046   Median : 7012   Median : 7008  
 Mean   :10088   Mean   : 8811   Mean   : 8767   Mean   : 8762  
 3rd Qu.:13630   3rd Qu.:11928   3rd Qu.:11870   3rd Qu.:11863  
 Max.   :40077   Max.   :34820   Max.   :34589   Max.   :34566  
  NonChimeric     BacteriaOnly       NoMock     
 Min.   :    6   Min.   :    6   Min.   :    6  
 1st Qu.: 4331   1st Qu.: 4322   1st Qu.: 4322  
 Median : 6580   Median : 6568   Median : 6568  
 Mean   : 8114   Mean   : 8108   Mean   : 8108  
 3rd Qu.:10737   3rd Qu.:10731   3rd Qu.:10731  
 Max.   :32147   Max.   :32142   Max.   :32142
```

## View the first 10 row of sequence length data

```
# A tibble: 10 x 7
   Original Screened Aligned Denoised NonChimeric BacteriaOnly NoMock
      <dbl>    <dbl>   <dbl>    <dbl>       <dbl>        <dbl>  <dbl>
 1       NA       NA      NA       NA          NA           NA     NA
 2      252      252     252      252         252          252    252
 3      252      252     252      252         252          252    252
 4      253      253     253      252         252          252    252
 5      253      155     252      252         252          252    252
 6      252      252     253      252         252          252    252
 7      124      252     252      253         253          253    253
 8      252      253     252      252         252          252    252
 9      253      252     253      253         253          253    253
10      253      252     211      253         253          253    253
```

---

## Graphical distribution of processed sequences

---

### Stacked barplot

> Note that it for large number of samples it is difficult to plot the x-axis. In such situation it is good to split the samples (see example below)

## Subsampling examples

> Below is a command for ssampling a specific dataset size. Here it shows how to filter samples with less that 2000 sequences.

### Samples with less than 2000 sequences

```
library(dplyr)
subsetlt2000 <- seqcount.v.m %>% as.data.frame() %>% dplyr::filter(value <2000)
```

  


---

### Barplots grouped by process

> Shows maximum sequence depth.

### Boxplots grouped by process

### Boxplots grouped by sex

### Boxplots grouped by time

  


---

### Density plots grouped by sex and time

  


---

### Histograms grouped by sex and time

---

## Publication-ready figure

- All subplots on single plot:

  


---

# Posible questions

### At sequence processing

- QN1: Do the paired sequences overlap as expected?
- QN2: Are there sequences to be removed from the analysis based on sequence length?
- QN3: Does the dorminant length match the targeted region of 16S rRNA gene?
- QN4: Are the representative sequences too few or too many? Note that too many non-redundant sequences may results from poor overlapping between forward and reverse reads. Review before proceeding to avoid misleading conclusions.

### At sequence classification

- QN1: What taxonomic classifier should be used? Think of need to develop and use custom classifier that suits the study objectives.
- QN2: Are there sequences assigned to non-bacterial taxonomic lineages?
- QN3: Should the matches to non-bacterial lineages be removed from further analysis?
- QN4: …….?

### At error estimation

- QN1: Is the error too high to cause rejection of the results results?
- QN2: …….?
- QN3: …….?
- QN4: …….?

  


---

## Summary of packages used in the analysis

```
R version 3.5.2 (2018-12-20)
Platform: x86_64-apple-darwin15.6.0 (64-bit)
Running under: macOS Mojave 10.14.4

Matrix products: default
BLAS: /Library/Frameworks/R.framework/Versions/3.5/Resources/lib/libRblas.0.dylib
LAPACK: /Library/Frameworks/R.framework/Versions/3.5/Resources/lib/libRlapack.dylib

locale:
[1] en_US.UTF-8/en_US.UTF-8/en_US.UTF-8/C/en_US.UTF-8/en_US.UTF-8

attached base packages:
[1] stats     graphics  grDevices utils     datasets  methods   base     

other attached packages:
[1] scales_1.0.0  ggpubr_0.2    magrittr_1.5  dplyr_0.8.0.1 ggplot2_3.1.0
[6] readr_1.3.1  

loaded via a namespace (and not attached):
 [1] Biobase_2.42.0      tidyr_0.8.3         jsonlite_1.6       
 [4] splines_3.5.2       foreach_1.4.4       assertthat_0.2.1   
 [7] highr_0.8           stats4_3.5.2        phyloseq_1.26.1    
[10] yaml_2.2.0          slam_0.1-45         pillar_1.3.1       
[13] lattice_0.20-38     glue_1.3.1          digest_0.6.18      
[16] XVector_0.22.0      colorspace_1.4-1    cowplot_0.9.4      
[19] htmltools_0.3.6     Matrix_1.2-15       plyr_1.8.4         
[22] tm_0.7-6            pkgconfig_2.0.2     microbiome_1.4.2   
[25] zlibbioc_1.28.0     purrr_0.3.2         tibble_2.1.1       
[28] mgcv_1.8-27         IRanges_2.16.0      withr_2.1.2        
[31] BiocGenerics_0.28.0 lazyeval_0.2.1      cli_1.1.0          
[34] NLP_0.2-0           survival_2.43-3     crayon_1.3.4       
[37] evaluate_0.13       fansi_0.4.0         nlme_3.1-137       
[40] MASS_7.3-51.1       xml2_1.2.0          vegan_2.5-4        
[43] tools_3.5.2         data.table_1.12.0   hms_0.4.2          
[46] stringr_1.4.0       Rhdf5lib_1.4.3      S4Vectors_0.20.1   
[49] munsell_0.5.0       cluster_2.0.7-1     Biostrings_2.50.2  
[52] ade4_1.7-13         compiler_3.5.2      rlang_0.3.4        
[55] rhdf5_2.26.2        grid_3.5.2          iterators_1.0.10   
[58] biomformat_1.10.1   igraph_1.2.4        labeling_0.3       
[61] rmarkdown_1.12      gtable_0.2.0        codetools_0.2-16   
[64] multtest_2.38.0     reshape2_1.4.3      iNEXT_2.0.19       
[67] R6_2.4.0            knitr_1.22          utf8_1.1.4         
[70] permute_0.9-4       ape_5.2             stringi_1.4.3      
[73] parallel_3.5.2      Rcpp_1.0.1          tidyselect_0.2.5   
[76] xfun_0.6
```
