# Supplementary material for: iMAP: an integrated bioinformatics and visualization pipeline for microbiome data analysis
Source: BMC Bioinformatics. 2019 Jul 3;20:374. doi: 10.1186/s12859-019-2965-4 (PMC6610863; doi:10.1186/s12859-019-2965-4)
Supplement: Supplementary file 5 — Preliminary analysis report generated automatically by the iMAP to provide a summary of conserved taxonomy assigned to OTUs and the initial analysis of OTUs and taxa data. The preliminary analysis report was automatically saved in the “reports” folder as “report4_preliminary_analysis.html”. (HTML 20379 kb) [file 12859_2019_2965_MOESM5_ESM.html]

 

 

 

 
 
 


 Progress report 4 

 
 
 
 
 
 
 
 
 

 
 
 


 

 


 


 

 

 

 


 

 


 


 


 Progress report 4 
 OTU clustering, Taxonomy assignment & Preliminary analysis 
 Updated: 2019-05-15 11:36:51 

 


   
 
 Conserved taxonomy assignment 
 
 Methods: 
 
 Phylotype method at genus level. 
 OTU-based method with minimum set at 97% identity. The opticlust algorithm was used to optimize the quality of assigned taxonomy. 
 Phylogeny method where tree was taxonomically classified with minimum set at 97% identity 
 
   
 
 
 Quality of conserved taxonomy 
 The opticlust algorithm yielded high quality OTUs based on the high precision metrics below. 
   
 
 Table x: Statistical parameters calculated from the OTU-based classification method 
 
 
 Parameter 
 Value 
 
 
 
 
 cutoff 
 0.2 
 
 
 Sensitivity 
 0.998 
 
 
 Specificity 
 0.999 
 
 
 PPV 
 0.998 
 
 
 NPV 
 0.999 
 
 
 Accuracy 
 0.999 
 
 
 MCC 
 0.997 
 
 
 F1score 
 0.998 
 
 
 FDR 
 0.002 
 
 
 
  -->
 
 
 
 Taxonomy assigned to observed OTUs 
 
 The taxonomy assignment is based on the phylotype method in mothur platform. Only taxon observed more than once are displayed. 
 
   
  -->
 
 
 
 Manual annotation 
 
 Integrating output obtained from diverse methods could be a robust way for profiling complex microbial communities present in a given sample. 
 The phylotype-based approach yielded 197 OTUs at genus level 
 The OTU-based approach generated 11,257 OTU clusters at 97% identity. 
 The phylogeny approach generated 58,929 tree nodes which were taxonomically classified at 80% identity. 
 Manual review and further annotation should lead to more insightful non-redundant results. 
 
   
   
 Figure x: Unfiltered and curated OTU abundance. Visual representation of taxon terms highlight the most abundant taxon based on frequency of being assigned to an OTU or tree nodes. Muribaculaceae is the most frequently assigned family and Muribaculaceae_ge is the most frequent species assigned to most sequences. 
 
 
   
 
 Most abundant species (top 15%) 
  
OTUs  
   [1] "Otu001" "Otu002" "Otu003" "Otu004" "Otu005" "Otu006" "Otu007"
 [8] "Otu008" "Otu009" "Otu010" "Otu012" "Otu013" "Otu014" "Otu015"
[15] "Otu016" "Otu017" "Otu020" "Otu023" "Otu027" "Otu032" "Otu036"
[22] "Otu038" "Otu042" "Otu045"  
  
Phylum  
  [1] "Bacteroidetes" "Firmicutes"     
  
Class  
  [1] "Bacilli"     "Bacteroidia" "Clostridia"   
  
Order  
  [1] "Bacteroidales"      "Bifidobacteriales"  "Clostridiales"     
[4] "Enterobacteriales"  "Erysipelotrichales" "Lactobacillales"   
[7] "Mollicutes_RF39"     
  
Family  
   [1] "Bacteroidaceae"      "Bifidobacteriaceae"  "Clostridiaceae_1"   
 [4] "Erysipelotrichaceae" "Family_XIII"         "Lachnospiraceae"    
 [7] "Lactobacillaceae"    "Mollicutes_RF39_fa"  "Muribaculaceae"     
[10] "Rikenellaceae"       "Ruminococcaceae"      
  
Genus  
   [1] "Alistipes"               "Bacteroides"            
 [3] "Bifidobacterium"         "Candidatus_Arthromitus" 
 [5] "Escherichia.Shigella"    "Family_XIII_ge"         
 [7] "Flavonifractor"          "Fusicatenibacter"       
 [9] "Lachnoclostridium"       "Lachnospiraceae_UCG.006"
[11] "Lactobacillus"           "Mollicutes_RF39_ge"     
[13] "Muribaculaceae_ge"       "Oscillospira"           
[15] "Roseburia"               "Ruminococcaceae_ge"     
[17] "Turicibacter"             
   
 
 
 Example of rank abundance 
 
 
 Demonstration using eight samples 
 Rank abundance curves can be used to display species richness and species evenness across selected samples. 
 
   
 Figure x. Rank abundance of eight samples. Package:  goeveg  
 
 
   
 
 
 Correlation between observed phyla 
 
 Seven different visualization methods can be used including:
 
 circle 
 square 
 ellipse 
 number 
 shade 
 color 
 pie 
 mixed 
 R package  corrplot . 
  
 
   
   
 Figure x. Correlation between species identified at phylum-level. Species are ordered alphabetically (top panel) and heuristically (bottom panel) 
 
 
   
 
 
 
 Alpha diversity 
   
 
 Species richness at each datapoint 
   
 Figure x: Stacked barplots for species richness. The estimated richness (green bars) was calculated using  chao  calculator and observed ichness (red bars) was calculated using  sobs . 
     
 
 
 Species richness: Boxplots, density plots and histograms 
   
 Figure x: Species richness (observed species) displayed by boxplot (A), density plots (B) and histograms (C). 
     
 
 
 Species richness plots 
 Include are: 
 
 Line plots: Points joined by straight lines 
 Scatter plot with correlation coefficient 
 Jitter boxplots 
 
   
 
 
   
 
 
 
 Species accumulation 
 
 
 Methods used
 
 Exact 
 Random 
 Collector 
 Rarefaction 
  
 
   
   
 
 Individual species accumulation curves using different methods 
   
   
 
 
 Overplot graph of species accumulation curves 
   
 
 
   
 
 
 
 Rarefaction and Extrapolation (R/E) 
 
 Type 1: Sample-size-based R/E curve 
   
 
 
   
 
 
 Type 2: Sample completeness curve 
   
 
 
 
   
 
 
 Type 3: Coverage‐based R/E curves 
   
   
 
 
 
   
 
 
 Shannon diversity index 
   
 
 
   
 
 
 Inverse Simpson diversity index 
   
  -->
 
 
   
 
 
 
 Beta diversity 
   
 
 
 Heatmaps 
 
 Phyla 
   
   
 
 
 Class 
   
   
 
 
 Order 
   
   
 
 
 Family 
   
   
 
 
 Genus 
   
   
 
 
 
 PAM clustering 
 
 
 Partitioning Around Medoids (PAM) 
 Is considered to be the more robust version of K-means. 
 Medoids are representative objects of the cluster. 
 Starts by determining the best number of clusters using factoextra::fviz_nbclust() 
 Method: Silhouette 
 Metric = Euclidean 
 Robust for partitioning data set into clusters of observation. 
 User are required to know the data to indicate the appropriate number of clusters to be produced. 
 Visualize clusters (pam results) using factoextra::fviz_cluster() 
 
   
 
 Number of best clusters 
   
  OTUbased  
  Number_clusters     Value_Index 
         2.0000         21.0681   
  Phylum  
  Number_clusters     Value_Index 
         2.0000         75.9057   
  Class  
  Number_clusters     Value_Index 
         2.0000         50.5688   
  Order  
  Number_clusters     Value_Index 
         2.0000         42.1392   
  Family  
  Number_clusters     Value_Index 
          3.000          30.029   
  Genus  
  Number_clusters     Value_Index 
         3.0000         20.7716   
   
 
 
 Visualization of best clusters 
   
 Figure x: Optimal number of OTU clusters. The suggested number of best clusters (dotted line) that could explain most variation is 2 for OTUs (A), 3 for phylum (B), 3 for class (C), 2 for Order (D), 10 for Family (E) and 2 for Genus (F). A high average Silhouette width indicates high quality clustering. 
   
 
 
 Cluster size 
 
 Using Silhouette Information 
 Use original data (non-matrices) 
 Measures how well a cluster is 
 Estimates the average distance between clusters. 
 
    cluster size ave.sil.width
1       1  240          0.65
2       2  120          0.13  
    cluster size ave.sil.width
1       1  240          0.68
2       2  120          0.24  
    cluster size ave.sil.width
1       1  239          0.67
2       2  121          0.22  
    cluster size ave.sil.width
1       1  239          0.67
2       2  121          0.22  
    cluster size ave.sil.width
1       1  241          0.67
2       2  119          0.10  
    cluster size ave.sil.width
1       1  235          0.68
2       2  125          0.23  
     
 
 
 Cluster validation 
   
 Figure x: Silhouette plot guided by the best number of clusters. Observations with a large Si (almost 1) are very well clustered. A small Si (around 0) means that the observation lies between two clusters while a negative Si are outliers. 
   
 
 
 Cluster visualization 
   
     
 
 
 
 Publication-ready plot for PAM-Silhouette clustering 
   
     
 
 
 PCA (Principal Component Analysis) 
 
 
 Identifies smaller number of uncorrelated variables (principal components) from a large set of data. 
 Explains the maximum amount of variance with the minimum number of principal components. 
 Missing values are replaced by the column mean 
 Use scree plot to estimate which components explain most of the variability in the data 
 
 
 Scree plot: Analysis of number of suitable components 
   
 Figure x: Scree plot of PCA. Shows which components explain most of the variability in the data. Over 80% of the variances contained in OTU and taxonomy data are retained by the first two principal components. The first PC explains the maximum amount of variation in the data set. 
   
 
 
 
 PCoA or PCO: Principal Coordinates Analysis 
 
 
   
 
 Barplots of PCoA Eigen Values 
   
   
 
 
 Point plots: PCoA Eigen Values 
        
 
   
 
 
 
 NMDS (Non-metric multidimensional scaling) 
 
 
 Goodness of fit and Sheperd plot can be used to determine the good or poor fit. 
 Stress values are used to indicate the quality of NMDS ordination, <0.1 are considered fair, <0.05 indicate good fit. 
  Reference  
 
  Square root transformation
Wisconsin double standardization
Run 0 stress 0.1320155 
Run 1 stress 0.131795 
... New best solution
... Procrustes: rmse 0.01737973  max resid 0.1721785 
Run 2 stress 0.1308199 
... New best solution
... Procrustes: rmse 0.03799815  max resid 0.2148348 
Run 3 stress 0.1309166 
... Procrustes: rmse 0.01927976  max resid 0.298676 
Run 4 stress 0.1321164 
Run 5 stress 0.1314121 
Run 6 stress 0.1310729 
... Procrustes: rmse 0.02276801  max resid 0.2876684 
Run 7 stress 0.1314575 
Run 8 stress 0.131648 
Run 9 stress 0.1309433 
... Procrustes: rmse 0.02510678  max resid 0.2631128 
Run 10 stress 0.1306583 
... New best solution
... Procrustes: rmse 0.01200417  max resid 0.1331687 
Run 11 stress 0.1315689 
Run 12 stress 0.1307628 
... Procrustes: rmse 0.01844118  max resid 0.2927609 
Run 13 stress 0.1305934 
... New best solution
... Procrustes: rmse 0.0275882  max resid 0.2930939 
Run 14 stress 0.1327801 
Run 15 stress 0.1308671 
... Procrustes: rmse 0.03324444  max resid 0.2022286 
Run 16 stress 0.1318062 
Run 17 stress 0.1312982 
Run 18 stress 0.1310071 
... Procrustes: rmse 0.02757587  max resid 0.2797129 
Run 19 stress 0.1305531 
... New best solution
... Procrustes: rmse 0.008945208  max resid 0.1223186 
Run 20 stress 0.1311254 
*** No convergence -- monoMDS stopping criteria:
    14: no. of iterations >= maxit
     6: stress ratio > sratmax  
  Square root transformation
Wisconsin double standardization
Run 0 stress 0.121395 
Run 1 stress 0.1226535 
Run 2 stress 0.1218715 
... Procrustes: rmse 0.01648084  max resid 0.1473789 
Run 3 stress 0.1226283 
Run 4 stress 0.1198947 
... New best solution
... Procrustes: rmse 0.01205629  max resid 0.08810511 
Run 5 stress 0.1204889 
Run 6 stress 0.1211368 
Run 7 stress 0.1243474 
Run 8 stress 0.1214427 
Run 9 stress 0.1208254 
Run 10 stress 0.1199683 
... Procrustes: rmse 0.009068018  max resid 0.09452289 
Run 11 stress 0.1225659 
Run 12 stress 0.1209852 
Run 13 stress 0.1198246 
... New best solution
... Procrustes: rmse 0.009211115  max resid 0.1108122 
Run 14 stress 0.1214443 
Run 15 stress 0.1212073 
Run 16 stress 0.1207379 
Run 17 stress 0.121377 
Run 18 stress 0.1224281 
Run 19 stress 0.1207474 
Run 20 stress 0.1204284 
*** No convergence -- monoMDS stopping criteria:
    15: no. of iterations >= maxit
     5: stress ratio > sratmax  
  Square root transformation
Wisconsin double standardization
Run 0 stress 0.1422875 
Run 1 stress 0.144234 
Run 2 stress 0.1430387 
Run 3 stress 0.1430329 
Run 4 stress 0.1432225 
Run 5 stress 0.1435328 
Run 6 stress 0.1435968 
Run 7 stress 0.1441753 
Run 8 stress 0.1438901 
Run 9 stress 0.1430608 
Run 10 stress 0.1426484 
... Procrustes: rmse 0.01034154  max resid 0.1433039 
Run 11 stress 0.1434003 
Run 12 stress 0.142515 
... Procrustes: rmse 0.004608976  max resid 0.05952907 
Run 13 stress 0.1444387 
Run 14 stress 0.1435266 
Run 15 stress 0.1446007 
Run 16 stress 0.1436055 
Run 17 stress 0.1435066 
Run 18 stress 0.143452 
Run 19 stress 0.1436154 
Run 20 stress 0.1511232 
*** No convergence -- monoMDS stopping criteria:
     8: no. of iterations >= maxit
    12: stress ratio > sratmax  
  Square root transformation
Wisconsin double standardization
Run 0 stress 0.1355536 
Run 1 stress 0.1355325 
... New best solution
... Procrustes: rmse 0.00733575  max resid 0.09385795 
Run 2 stress 0.1443072 
Run 3 stress 0.1386428 
Run 4 stress 0.135253 
... New best solution
... Procrustes: rmse 0.008139862  max resid 0.1145029 
Run 5 stress 0.1357678 
Run 6 stress 0.1428662 
Run 7 stress 0.1366844 
Run 8 stress 0.1351755 
... New best solution
... Procrustes: rmse 0.00813885  max resid 0.1237319 
Run 9 stress 0.1382688 
Run 10 stress 0.1355883 
... Procrustes: rmse 0.007218701  max resid 0.07368605 
Run 11 stress 0.1354217 
... Procrustes: rmse 0.006385372  max resid 0.09222482 
Run 12 stress 0.1355949 
... Procrustes: rmse 0.007018928  max resid 0.09511988 
Run 13 stress 0.1396045 
Run 14 stress 0.1359311 
Run 15 stress 0.1353594 
... Procrustes: rmse 0.004678551  max resid 0.04709641 
Run 16 stress 0.1360675 
Run 17 stress 0.1365917 
Run 18 stress 0.1354206 
... Procrustes: rmse 0.005836932  max resid 0.06944482 
Run 19 stress 0.1361279 
Run 20 stress 0.1356546 
... Procrustes: rmse 0.007671726  max resid 0.0759837 
*** No convergence -- monoMDS stopping criteria:
    17: no. of iterations >= maxit
     3: stress ratio > sratmax  
  Square root transformation
Wisconsin double standardization
Run 0 stress 0.1432815 
Run 1 stress 0.1429304 
... New best solution
... Procrustes: rmse 0.01350941  max resid 0.1931366 
Run 2 stress 0.1459068 
Run 3 stress 0.1429461 
... Procrustes: rmse 0.007475489  max resid 0.09080252 
Run 4 stress 0.1450437 
Run 5 stress 0.1444651 
Run 6 stress 0.1458861 
Run 7 stress 0.1450571 
Run 8 stress 0.1434276 
... Procrustes: rmse 0.009615488  max resid 0.1266274 
Run 9 stress 0.1432483 
... Procrustes: rmse 0.008113319  max resid 0.115036 
Run 10 stress 0.1439801 
Run 11 stress 0.1441929 
Run 12 stress 0.1435686 
Run 13 stress 0.1429727 
... Procrustes: rmse 0.006387782  max resid 0.09585337 
Run 14 stress 0.145977 
Run 15 stress 0.142738 
... New best solution
... Procrustes: rmse 0.008637825  max resid 0.09908415 
Run 16 stress 0.1488512 
Run 17 stress 0.1453525 
Run 18 stress 0.1429162 
... Procrustes: rmse 0.006302938  max resid 0.07125081 
Run 19 stress 0.143163 
... Procrustes: rmse 0.008844548  max resid 0.1058869 
Run 20 stress 0.1436694 
*** No convergence -- monoMDS stopping criteria:
    17: no. of iterations >= maxit
     3: stress ratio > sratmax  
  Square root transformation
Wisconsin double standardization
Run 0 stress 0.1483427 
Run 1 stress 0.1481365 
... New best solution
... Procrustes: rmse 0.03931585  max resid 0.6391495 
Run 2 stress 0.1477953 
... New best solution
... Procrustes: rmse 0.03100381  max resid 0.4892752 
Run 3 stress 0.1496372 
Run 4 stress 0.1495392 
Run 5 stress 0.1480876 
... Procrustes: rmse 0.01591379  max resid 0.249 
Run 6 stress 0.1480336 
... Procrustes: rmse 0.02721307  max resid 0.4228015 
Run 7 stress 0.1479726 
... Procrustes: rmse 0.01052854  max resid 0.1656784 
Run 8 stress 0.150896 
Run 9 stress 0.1482884 
... Procrustes: rmse 0.022387  max resid 0.33595 
Run 10 stress 0.1482097 
... Procrustes: rmse 0.03535344  max resid 0.564696 
Run 11 stress 0.1480284 
... Procrustes: rmse 0.02688119  max resid 0.4016029 
Run 12 stress 0.1485284 
Run 13 stress 0.1490377 
Run 14 stress 0.1479164 
... Procrustes: rmse 0.009144612  max resid 0.137554 
Run 15 stress 0.1482056 
... Procrustes: rmse 0.03356324  max resid 0.5510062 
Run 16 stress 0.1488457 
Run 17 stress 0.1480399 
... Procrustes: rmse 0.01489924  max resid 0.2183789 
Run 18 stress 0.147993 
... Procrustes: rmse 0.02910497  max resid 0.4506213 
Run 19 stress 0.1490598 
Run 20 stress 0.147806 
... Procrustes: rmse 0.01093966  max resid 0.1569197 
*** No convergence -- monoMDS stopping criteria:
    11: no. of iterations >= maxit
     9: stress ratio > sratmax  
   
 
 Parameters used for the NMDS 
  
OTUs
----------------------------  
  
Call:
metaMDS(comm = otu.t, distance = "bray", k = 3, try = 10, display = c("sites"),      choices = c(1, 2), type = "t", shrink = FALSE) 

global Multidimensional Scaling using monoMDS

Data:     wisconsin(sqrt(otu.t)) 
Distance: bray 

Dimensions: 3 
Stress:     0.1305531 
Stress type 1, weak ties
No convergent solutions - best solution after 20 tries
Scaling: centring, PC rotation, halfchange scaling 
Species: expanded scores based on 'wisconsin(sqrt(otu.t))'   
  
Phylum
----------------------------  
  
Call:
metaMDS(comm = phylum.t, distance = "bray", k = 3, try = 10,      display = c("sites"), choices = c(1, 2), type = "t", shrink = FALSE) 

global Multidimensional Scaling using monoMDS

Data:     wisconsin(sqrt(phylum.t)) 
Distance: bray 

Dimensions: 3 
Stress:     0.1198246 
Stress type 1, weak ties
No convergent solutions - best solution after 20 tries
Scaling: centring, PC rotation, halfchange scaling 
Species: expanded scores based on 'wisconsin(sqrt(phylum.t))'   
  
Class
----------------------------  
  
Call:
metaMDS(comm = class.t, distance = "bray", k = 3, try = 10, display = c("sites"),      choices = c(1, 2), type = "t", shrink = FALSE) 

global Multidimensional Scaling using monoMDS

Data:     wisconsin(sqrt(class.t)) 
Distance: bray 

Dimensions: 3 
Stress:     0.1422875 
Stress type 1, weak ties
No convergent solutions - best solution after 20 tries
Scaling: centring, PC rotation, halfchange scaling 
Species: expanded scores based on 'wisconsin(sqrt(class.t))'   
  
Order
----------------------------  
  
Call:
metaMDS(comm = order.t, distance = "bray", k = 3, try = 10, display = c("sites"),      choices = c(1, 2), type = "t", shrink = FALSE) 

global Multidimensional Scaling using monoMDS

Data:     wisconsin(sqrt(order.t)) 
Distance: bray 

Dimensions: 3 
Stress:     0.1351755 
Stress type 1, weak ties
No convergent solutions - best solution after 20 tries
Scaling: centring, PC rotation, halfchange scaling 
Species: expanded scores based on 'wisconsin(sqrt(order.t))'   
  
Family
----------------------------  
  
Call:
metaMDS(comm = family.t, distance = "bray", k = 3, try = 10,      display = c("sites"), choices = c(1, 2), type = "t", shrink = FALSE) 

global Multidimensional Scaling using monoMDS

Data:     wisconsin(sqrt(family.t)) 
Distance: bray 

Dimensions: 3 
Stress:     0.142738 
Stress type 1, weak ties
No convergent solutions - best solution after 20 tries
Scaling: centring, PC rotation, halfchange scaling 
Species: expanded scores based on 'wisconsin(sqrt(family.t))'   
  
Genus
----------------------------  
  
Call:
metaMDS(comm = genus.t, distance = "bray", k = 3, try = 10, display = c("sites"),      choices = c(1, 2), type = "t", shrink = FALSE) 

global Multidimensional Scaling using monoMDS

Data:     wisconsin(sqrt(genus.t)) 
Distance: bray 

Dimensions: 3 
Stress:     0.1477953 
Stress type 1, weak ties
No convergent solutions - best solution after 20 tries
Scaling: centring, PC rotation, halfchange scaling 
Species: expanded scores based on 'wisconsin(sqrt(genus.t))'   
   
 
 
 Sherperd and non-metric multidimensional scaling plots. 
   
 
 
   
 Figure X. Sherperd and non-metric multidimensional scaling plot. Green oints represent samples and red points represent OTU or species. Similar samples are ordinated together. Stress values are shown at the botthom of ordination plot. 
     
 
 
 
 Phylogenetic clustering and tree annotation 
 
   
 Figure x: The circular phylograms ( A ), unrooted cladogram ( B ), and the rectangular phylograms ( C ) display the relationships of the 360 samples used in the case study. Female (red) and male (blue) linked with sequence counts showing the proportion of the number of classified (green) and unclassified (red) displayed on a pie chart followed by the phyla abundance (heatmap) and species richness bar chart showing the observed (green) and estimated (maroon) richness. A portion of the tree ( D ) is enlarged to show some details. 
 
 
 Posible questions 
 
 
 Alpha diversity 
 
 QN1: Are the values obtained too sensitive to sampling? 
 QN2: Was the sampling effort sufficient to account for most OTUs present in a sample? 
 QN3: Is there a need to continue with re-sampling? 
 QN4: …….? 
 
   
 
 
 Beta diversity 
 
 QN1: …….? 
 QN2: …….? 
 QN3: …….? 
 QN4: …….? 
 
   
 
 
 
 More intervention by investigators 
 
 Testing hypotheses statistically …… 
 Conducting biological-based analysis and intepretation …… 
 Conducting focused annotation …… 
 ………. 
 ………. 
 
   
 
 Summary of packages used in the analysis 
  R version 3.5.2 (2018-12-20)
Platform: x86_64-apple-darwin15.6.0 (64-bit)
Running under: macOS Mojave 10.14.4

Matrix products: default
BLAS: /Library/Frameworks/R.framework/Versions/3.5/Resources/lib/libRblas.0.dylib
LAPACK: /Library/Frameworks/R.framework/Versions/3.5/Resources/lib/libRlapack.dylib

locale:
[1] en_US.UTF-8/en_US.UTF-8/en_US.UTF-8/C/en_US.UTF-8/en_US.UTF-8

attached base packages:
[1] grid      stats     graphics  grDevices utils     datasets  methods  
[8] base     

other attached packages:
 [1] factoextra_1.0.5 cluster_2.0.7-1  NbClust_3.0      iNEXT_2.0.19    
 [5] goeveg_0.4.2     vegan_2.5-4      permute_0.9-4    scales_1.0.0    
 [9] ggpubr_0.2       magrittr_1.5     dplyr_0.8.0.1    reshape2_1.4.3  
[13] funModeling_1.7  Hmisc_4.2-0      ggplot2_3.1.0    Formula_1.2-3   
[17] survival_2.43-3  lattice_0.20-38 

loaded via a namespace (and not attached):
 [1] colorspace_1.4-1     htmlTable_1.13.1     XVector_0.22.0      
 [4] base64enc_0.1-3      rstudioapi_0.10      ggrepel_0.8.0       
 [7] xml2_1.2.0           leaps_3.0            codetools_0.2-16    
[10] splines_3.5.2        knitr_1.22           ade4_1.7-13         
[13] spam_2.2-1           jsonlite_1.6         entropy_1.2.1       
[16] phyloseq_1.26.1      readr_1.3.1          compiler_3.5.2      
[19] backports_1.1.3      assertthat_0.2.1     Matrix_1.2-15       
[22] lazyeval_0.2.1       acepack_1.4.1        htmltools_0.3.6     
[25] tools_3.5.2          igraph_1.2.4         dotCall64_1.0-0     
[28] NLP_0.2-0            gtable_0.2.0         glue_1.3.1          
[31] FactoMineR_1.41      maps_3.3.0           Rcpp_1.0.1          
[34] slam_0.1-45          Biobase_2.42.0       Biostrings_2.50.2   
[37] multtest_2.38.0      gdata_2.18.0         ape_5.2             
[40] nlme_3.1-137         iterators_1.0.10     xfun_0.6            
[43] stringr_1.4.0        gtools_3.8.1         zlibbioc_1.28.0     
[46] MASS_7.3-51.1        hms_0.4.2            parallel_3.5.2      
[49] biomformat_1.10.1    rhdf5_2.26.2         RColorBrewer_1.1-2  
[52] fields_9.6           yaml_2.2.0           gridExtra_2.3       
[55] pander_0.6.3         rpart_4.1-13         latticeExtra_0.6-28 
[58] stringi_1.4.3        S4Vectors_0.20.1     corrplot_0.84       
[61] foreach_1.4.4        checkmate_1.9.1      caTools_1.17.1.1    
[64] BiocGenerics_0.28.0  rlang_0.3.4          pkgconfig_2.0.2     
[67] moments_0.14         bitops_1.0-6         evaluate_0.13       
[70] ROCR_1.0-7           purrr_0.3.2          Rhdf5lib_1.4.3      
[73] htmlwidgets_1.3      labeling_0.3         cowplot_0.9.4       
[76] tidyselect_0.2.5     plyr_1.8.4           R6_2.4.0            
[79] IRanges_2.16.0       gplots_3.0.1.1       pillar_1.3.1        
[82] foreign_0.8-71       withr_2.1.2          mgcv_1.8-27         
[85] scatterplot3d_0.3-41 nnet_7.3-12          tibble_2.1.1        
[88] crayon_1.3.4         KernSmooth_2.23-15   microbiome_1.4.2    
[91] rmarkdown_1.12       data.table_1.12.0    flashClust_1.01-2   
[94] digest_0.6.18        tm_0.7-6             tidyr_0.8.3         
[97] stats4_3.5.2         munsell_0.5.0         
 
 


 

 

 
 

 
 
